# Playing nice in the sandbox: On the role of heterogeneity, trust and cooperation in common-pool resources

**DOI:** 10.1371/journal.pone.0237870

**Published:** 2020-08-28

**Authors:** Fijnanda van Klingeren

**Affiliations:** Nuffield College, University of Oxford, Oxford, United Kingdom; Middlesex University, UNITED KINGDOM

## Abstract

The increasing heterogeneity of populations affects cooperation in common-pool resources in a time where the depletion of natural resources is a growing problem. This study investigates the effects of economic and sociocultural heterogeneity on trust and cooperation in common-pool resources using a laboratory experiment. The experiment comprises two Investment Games and a Common-Pool Resource Game, with a sample of 344 subjects from the United Kingdom and the Netherlands. By measuring the effects of economic and sociocultural heterogeneity separately as well as combined, this study disentangles the effects of specific heterogeneity types on cooperation in common-pool resources; something that has not been done before. Higher levels of trusting behaviour are found to have a positive effect on cooperation on the micro- and macro-level over time. While theory suggests negative effects of both forms of heterogeneity on cooperation through decreased levels of trust, the results show a surprising positive effect of economic heterogeneity on cooperation, but a negative effect if economic and sociocultural heterogeneity are combined. This study concludes that economic inequality can promote cooperation in CPRs, unless this inequality is lined up with sociocultural differences.

## Introduction

Societies are becoming more diverse on ethnic, cultural and economic dimensions due to growing migrant populations all over the world, especially in Northern Africa, Western Asia and Sub-Saharan Africa [1]. This increasing heterogeneity may pose a challenge for the successful management of common-pool resources [CPRs] as it can diversify interests and decrease trust between appropriators [2]. Especially when there are multiple larger groups of different sociocultural backgrounds living together, intergroup antagonism becomes stronger and coordination between groups becomes harder [3]. The deforestation of tropical forests and overfishing of the seas are examples of appropriators failing to work together on the preservation of natural resources. However, how and to what extent economic and sociocultural heterogeneity, and importantly the combination of the two, affect cooperation is still contested [4–12]. In particular, experimental research looking into the effect of heterogeneity on cooperation in CPRs is still relatively rare [13]. The aim of this paper is to provide insights into the relation between economic and sociocultural heterogeneity and sustainable cooperation in CPRs, both on the individual and the collective level. To do so, this study employs an Investment Game and a CPR game in a computerised laboratory experiment. Since part of the theoretical mechanism is often suggested to be the negative influence of heterogeneity on trust [14–18] and the beneficial influence of trust on society [19–25], this paper will consider trust as an important variable in the theoretical framework. Disentangling effects of economic and sociocultural heterogeneity using experiments in CPR settings has, to the extent of my knowledge, never been done before.

Two key characteristics of CPRs are their non-excludability and high subtractability: it is hard to exclude potential users from accessing CPRs, and the resource may run out [26]. These characteristics make CPRs vulnerable to the ‘tragedy of the commons’ as famously described by Hardin [27]: a situation in which the CPR is locked into a system that provides each resource-user incentives to use the limited resource unlimitedly, which will lead to its inevitable decay. From a game theoretic perspective, this tragedy will always take place, as a (myopic) rational individual will free-ride and overexploit the resource despite the long-term benefits of cooperation. Although there are plenty of examples of unsustainable appropriation of CPRs (see for instance [28, 29]), Hardin’s prediction is challenged by evidence from field research suggesting that CPR users are able to self-organise using institutions for collective action [30, 31]. In addition, an extensive body of research suggests more complex behaviour than predicted by rational choice theory [13, 29, 32–35]. With examples of both successful and failed CPRs, it is interesting and important to investigate the role that economic and sociocultural heterogeneity in communities surrounding CPRs play in the success or failure of a CPR.

Empirically analysing the influence of heterogeneity in real-life CPR settings can be a challenge due to the number of confounding factors that influence success and failure; one can never be entirely sure that one variable or a set of variables caused an outcome [36]. This can be solved to a large extent by using laboratory experiments. While laboratory experiments score lower on external validity than field experiments, they score high on internal validity: they allow one to test causality by measuring the impact of an isolated variable or set of variables repeatedly, in the same controlled setting—something that is not possible in field research [36, 37].

For the current study, subjects first played an Investment Game [IG] to measure general trust and trustworthiness and then played a CPR game as introduced by Janssen, Holahan and Ostrom [31], to measure behaviour in a CPR setting under different levels of heterogeneity. The CPR game mimics a fishing ground which the subjects can appropriate in return for money in groups of four under different conditions of economic and sociocultural heterogeneity. Economic heterogeneity is introduced as unequal endowments of players, and sociocultural heterogeneity by means of a Minimal Group Experiment [MGE]. An MGE is a method to create artificial identities based on a trivial criterion (see amongst others [38–41]). An extensive explanation of the MGE used in this paper is given in the section on experimental treatments.

Given the increasing depletion of CPRs world-wide and the rising levels of sociocultural and economic heterogeneity, the subject of heterogeneity and sustainable cooperation is gaining importance. The results of this study may be relevant not only for classic resources such as fishing grounds, but also for the growing number of contemporary commons such as food cooperatives and green energy initiatives [30, 42, 43].

The paper is structured as follows. First, existing literature on the research topic will be explored and hypotheses based on the literature are derived. Second, the experimental proceedings, games and treatments will be described. Third, descriptive plots, non-parametric test and multilevel regression are employed to analyse the data. Lastly, expectations are revisited, the findings are discussed and a conclusion is formulated.

## Existing literature

### Heterogeneity and cooperation

Experimental research looking into the effects of economic and sociocultural heterogeneity on cooperation suggests that asymmetrical endowments—i.e. an unequal division of money or points to spend between players in a group—lead to unequal contributions, unequal payoffs, and Pareto suboptimal outcomes [44]. Furthermore, it is argued that economic inequality leads to an increase of transaction costs [25] and diversification of interests among individuals, which makes cooperation less likely to happen [45–47]. For Public Good games it was found that heterogeneity in endowments indeed leads to a lower contribution to the public good [13, 35, 48]. This may be caused by an “anticipated reciprocity” effect: a situation in which subjects with a lower endowment expect the subjects with higher endowments to invest more, since they have more means available to invest, while subjects with higher endowments do not in fact do so [13].

Olson [9] suggests an opposite effect of economic heterogeneity: he argues that when groups are sufficiently small, and inequality sufficiently large, economic inequality leads to inequality of incentives, which makes the rich incentivised enough to bear the burden of cooperation by themselves. However, Bardhan and Dayton-Johnson [6] argue that this will only hold if there are “non-convexities” in the CPR, such as maintenance of the CPR, restraints on appropriation or large start-up costs. Most case-study literature still suggests a negative effect of economic heterogeneity [49, 50], despite the theoretical possibility as sketched by Olson.

Theory on the relation between sociocultural heterogeneity and cooperation suggests that individuals are more likely to cooperate with others from their ingroup: individuals with whom they share strong, multi-stranded relationships and common interests [15, 26, 51, 52–60]. Several case studies show that heterogeneity between CPR appropriators in terms of ethnicity, use of the resource, and view on the resource can be cause for conflict and hampers the development of regulation [29, 61]. Experimental research shows that (induced) group identity leads to positive behaviour towards ingroup members relative to outgroup members [62] and to the prioritising of group interest over individual interests [63]. Timilsina, Kotani and Kamijo [64] show in their research on sustainability of CPRs that subjects from urban areas show less prosocial and sustainable behaviour in a CPR game than subjects from rural areas. They suggest that subjects from urban areas are less prosocial as they come from a more heterogeneous and anonymous environment, whereas subjects from rural areas are more homogeneous and have a long tradition of necessary mutual cooperation. Habyarimana, Humphreys, Posner and Weinstein [65] suggest that people with the same ethnicity cooperate due to an increased “findability” in the ingroup through norms and tight social networks, and thus a higher probability of being punished for defection. Their research, identifying subjects as specific player “types”, suggests that homogeneity increases cooperation levels even for player types that are least likely to cooperate. Next to this, research shows that people have strong expectations of cooperation when interacting with ingroup members as opposed to outgroup members, which makes them more likely to cooperate themselves [66, 67]. However, Yamagishi and Kiyonari [67] show that in order for people to have these higher expectations of cooperation and to act upon it, it is necessary for players to 1) know the ingroup or outgroup identity of the other players; and 2) to know that the other players are aware that everyone knows everyone’s identity.

There is also research illustrating that sociocultural heterogeneity does not always have a negative impact on cooperation. Gehrig, Schlüter and Hammerstein [68] for instance, show in their study of fishermen from a small-scale fishery in Zanzibar that heterogeneous groups of fishermen from different villages do not cooperate less than homogeneous groups, despite a history of conflict between the villages. They argue that the effect of sociocultural heterogeneity on cooperation may be dependent on the institutional scope in the economic domain. In addition, Varughese and Ostrom [60] argue that heterogeneity does not influence cooperation when the right institutional arrangements are in place. However, the ease with which institutional arrangements can be set in place may depend on the type of heterogeneity. Bazzi, Gaduh, Rothenberg and Wong [3] show in their study of a population resettlement program in Indonesia that in the context of polarisation (a situation with a few larger groups with different sociocultural backgrounds) public goods provision is reduced, the likelihood of ethnic conflict is increased and economic development is hampered. In the context of fractionalisation (a situation with many smaller groups) these negative effects of sociocultural heterogeneity are not found.

### Trust

To get a better understanding on how heterogeneity affects cooperation, trust is considered to be a mediating variable. There is extensive evidence that heterogeneity reduces trust [14, 69–72], while higher trust yields higher levels of cooperation [2, 20, 21, 73]. This implies that individuals trust others with a similar identity—for instance religion, ethnicity, culture, social identity or something else—more than others with a different identity [14, 16, 18, 20, 24, 25, 74].

In the particular case that is studied here—a fishing ground—trust is necessary to maintain cooperation. Real fishermen do not know how much fish the other fishermen are catching during a day out fishing at sea, and will only see or hear about each other’s catch when all the ships have returned. In the same way, players in the experiment do not know what the others are doing during the appropriation stage and only receive information about this at the end of the period. Like fishermen, players will have to trust each other to behave cooperatively during the appropriation stage due to a lack of mutual monitoring.

### Hypotheses

Based on the majority of the discussed literature, the following hypothesis is deduced with regard to the direct effect of heterogeneity on cooperation in CPRs:

**Hypothesis 1** *(a) Economic and (b) sociocultural heterogeneity have a negative direct effect on cooperation over time and (c) the coincidence of economic and sociocultural heterogeneity has an even stronger negative direct effect on cooperation over time*.

The following hypotheses are deduced with regard to the indirect effect of heterogeneity on cooperation in CPRs, through trust:

**Hypothesis 2** *(a) Economic and (b) sociocultural heterogeneity have a negative effect on trust and (c) the coincidence of economic and sociocultural heterogeneity has an even stronger negative effect on trust*.

**Hypothesis 3** *Trust has a positive effect on cooperation over time*.

## Materials and methods

### Experimental sessions

A computerised laboratory experiment was designed and programmed in z-tree [75]. A total of 344 subjects of age 18 and older participated in the experiment, which was conducted at the Centre for Experimental Social Sciences [CESS] at Nuffield College, University of Oxford between October 2018 and November 2019, and at the Experimental Laboratory for Sociology and Economics [ELSE] at Utrecht University in April 2019. The subjects for both laboratories were recruited from the Online Recruitment System for Economic Experiments [ORSEE] [76]. After a pre-test with 16 Oxford students, the experiment was held in 13 sessions at CESS containing 248 subjects, and 5 sessions at ELSE containing 96 subjects. Sessions contained 16, 20 or 24 subjects. 81% of the subjects were students, from varying disciplines and years/stages. 60% of the subjects were female, and the average age was 26. This research, including the pre-test, has obtained ethics clearance from the Research Ethics Committee of Department of Sociology (DREC) at the University of Oxford (Ref. SOC_R2_001_C1A_18_30), the CESS ethics committee (Ref. LE_0044) and was covered by the ethical approval (Ref. FETC17-028, Buskens) of the ethical committee of the Faculty of the Social and Behavioural Sciences at Utrecht University. Written consent was obtained from all subjects in the study, including the pre-test, before the start of each experimental session. The data was anonymised before the analyses.

The laboratories are very similar in physical setup: a room with 25 to 30 computers, separated by privacy dividers, with a designated area for the experimenter computer. The experimental procedures, including having participants wait in a waiting room, dividing subjects randomly over computers, and handing out instructions with help of a research assistant, were formalised in the exact same way for sessions in the UK and the Netherlands.

**Fig 1:**
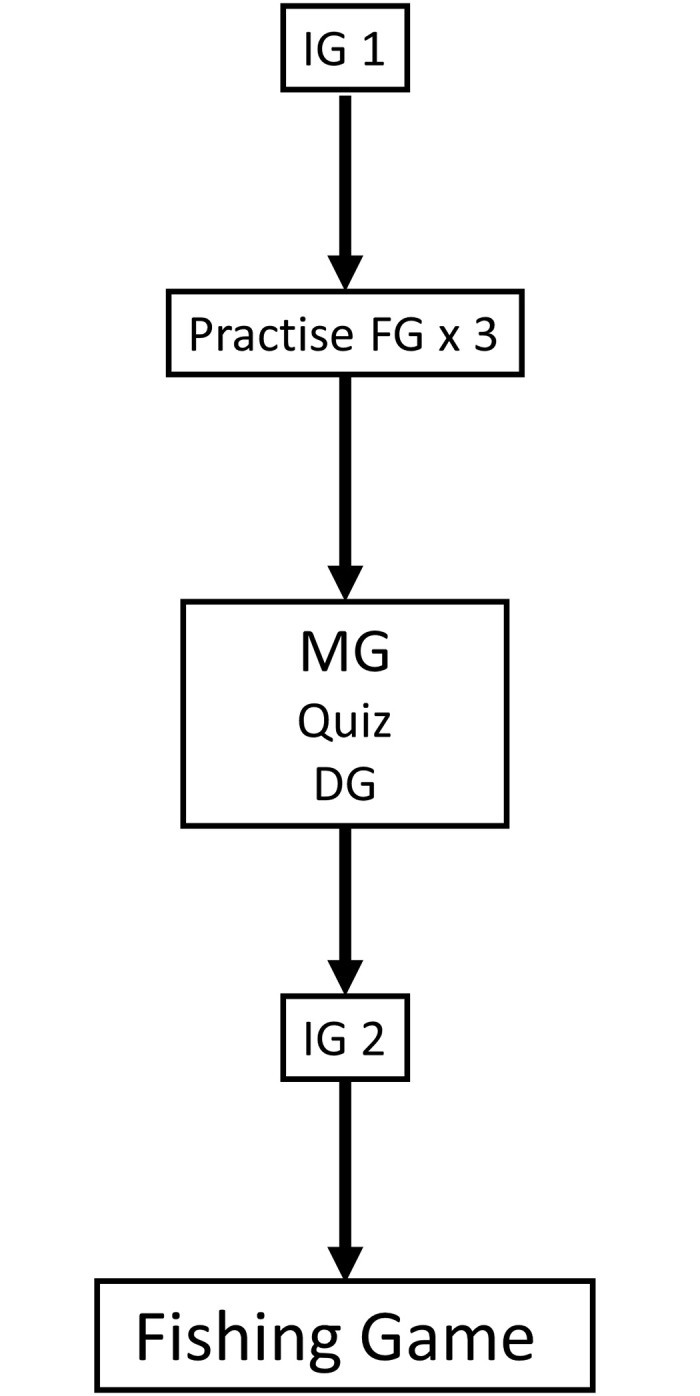
Course of the experiment.

The sequence of the experiment is as follows. When subjects enter the lab, they are given general written instructions in English for the first part of the experiment, which entails (1) a one-shot Investment Game; (2) a basic, practise version of the Fishing Game without any treatments for 3 periods; (3) a Minimal Group Experiment; (4) a binary other-other Dictator Game; and (5) a quiz to strengthen group bonds; and lastly (6) two other one-shot rounds of the Investment Game, once with an ingroup member and once with an outgroup member. An extensive explanation of each of these parts is provided in the upcoming sections. After completing the first part of the experiment, subjects receive instructions for the second part of the experiment: the Fishing Game with heterogeneity treatments. Subjects receive instructions specific to their treatment; economic heterogeneity [EH], sociocultural heterogeneity [SH], economic and sociocultural heterogeneity [EHSH] and the control treatment with no heterogeneity [NH]. The general and treatment-specific instructions can be found in S1 and S2 Text respectively. Completing the experiment took 50 to 70 minutes, of which 20 to 30 minutes were spent on the games before the Fishing Game. Fig 1 shows a schematic overview of the separate parts of the experiment. Subjects played for real money (GBP in the UK and EUR in the Netherlands) under an exchange rate of 500 units = 1 GBP/EUR. The average earning was 15.11 GBP/EUR.

### The Investment Game

The game that is used to measure trust before the main experiment is a variation of an Investment Game, as designed by Berg, Dickhaut and McCabe [77]. The Investment Game [IG], also called the Trust Game, is the most frequently used game to study trust [78].

The current version of the game is played as follows. There are two roles: the sending and the receiving role. Both players first adopt the sending role, and are given an endowment of 10 points. Both players are given the choice of sending points to another player, ranging from 0 to 10 points. This amount will be tripled once before it reaches the other player. Next, both players are put in the receiving role; they are asked how many of the points received by a sending player they would send back, for every possible amount of points received. This amount ranges from 0 to 30 points in increments of 3. 30 points would be the maximum amount to be received by the receiving player, since the maximum amount of points that the sending player can send is 10, and 3 × 10 = 30. Making players choose how many points to return for every possible received amount is called the strategy method [79, 80]. This method provides the advantage to the researcher of having data on trust and trustworthiness for all subjects, as the player 2 decision can be made separately from another subject’s player 1 decision. After playing the game in each of the roles, the subjects are randomly assigned the role for which they will receive their payoff. They are matched to another player with the other role. Their previous actions decide their final payoff. The material utility functions for the players are as follows: For player 1, the sender/trustor, the general material utility payoff function is:

$$\begin{array}{c} U_{i}=E_{i}-S_{ij}+R_{ji} \end{array}$$

Where *E*_*i*_ is the initial endowment of sender *i*, *S*_*ij*_ is the amount of points sent from the sender *i* to the receiver *j*, and *R*_*ji*_ is the amount of points returned from the receiver to the sender. For player 2, the receiver/trustee, the general material utility function is:

$$\begin{array}{c} U_{j}=E_{j}+3\times S_{ij}-R_{ji} \end{array}$$

Where *E*_*j*_ is the initial endowment of receiver *j*, *S*_*ij*_ is the amount of points sent from the sender *i* to the receiver *j* that is multiplied by 3 by the experimenter, and *R*_*ji*_ is the returned amount from the receiver to the sender. In the current game, *E* = 10 for both players. The two trust variables, operationalised following Johnson and Mislin [80] will be measured as follows:

$$\begin{array}{c} Trust=\frac{number\;of\;points\;sent\;by\;i}{endowment\;of\;i}=\frac{S_{ij}}{Ei} \end{array}$$

$$\begin{array}{c} Trustworthiness=\frac{number\;of\;points\;returned\;to\;i\;by\;j}{number\;of\;points\;available\;to\;return\;to\;i\;by\;j}=\frac{R_{ji}}{E_{j}+3\times S_{ij}} \end{array}$$

A graphic representation of an interaction between two matched players in the Investment Game is presented in S1 Fig. Half of the subjects will be paid for the sending role, and half for the receiving role in each Investment Game. Trustworthiness for all subjects will be calculated as the average trustworthiness over the 10 decisions every subject makes as the receiving player, as facilitated by using the strategy method. There are many variations of the Investment Game. A list and explanation of the specific characteristics of the Investment Game used in this experiment can be found in S3 Text.

### The Fishing Game

The game to be played by participants of the experiment is a CPR game. While it is common to measure cooperation, trust and heterogeneity in games such as Public Good [PG] games (see for instance Aksoy [81]), there are fundamental differences between CPRs and PGs that should be taken into account when looking specifically at CPR situations. Two characteristic features of a CPR situation are that exclusion of the collective good is infeasible—for instance, it is very costly to fence off part of an ocean—and that subtractability is high—the resource is finite and can run out [26, 29]. Table 1 shows the classification of different types of goods as shown by Ostrom, Walker and Gardner [26].

**Table 1:** A classification of goods (Ostrom et al., 1992, p.7).

		Subtractability	
		Low	High
Exclusion	Difficult	Public Goods	common-pool Resources
	Easy	Toll Goods	Private goods

The CPR in the current game is a fishing ground. In the game, there are four appropriators that use the CPR, who will play the game with each other for the entire session. All subjects first practise the basic CPR game without treatments for three periods, without any consequences for their payoff, to get to know the game. The real game with treatments is played for 40 periods. A time span of 40 periods is long enough for subjects to see the resource fall into decay if they overexploit it systematically, and there is enough time for subjects to adjust their investments to regrow the resource again.

#### Appropriation of the resource

At the beginning of each period *t*, the appropriators all receive an endowment *E* of units to invest in appropriation of the resource, *R*. Since appropriation of the CPR is a costly activity—e.g. it takes time and requires maintenance of the boat and fishing nets—the appropriation effort *a*, where 0 ≤ *a* ≤ *E*, represents the amount of effort an appropriator can invest in appropriation of the CPR.

The actors can choose how much they want to invest in appropriation of the resource each period. All appropriators make their appropriation choice at the same time, without knowing what the other appropriators do that period. They see how many fish there are in the lake and how many fish they receive per invested unit *a*. The appropriators all receive the same return $\left(\frac{4}{R_{0}}R_{t-1}\right)$ per appropriation effort unit of *a*. The material utility function for the appropriators per period is as follows:

$$\begin{array}{c} U_{it}=(\frac{4}{R_{0}}R_{t-1})a_{it}+(E_{i}-a_{it}) \end{array}$$

*U*_*it*_ is the total material utility of an appropriator *i* at timepoint *t*. In the function, *a*_*it*_ is the invested appropriation effort of appropriator *i* at timepoint *t*, and *E*_*i*_ is the endowment of appropriator *i*, which is the same every period. *R*_0_ is the original resource size of the CPR (i.e. the maximum number of fish in the lake) for which *R*_0_ = 600 is taken. *R*_*t*−1_ is the resource size at time *t* − 1. The profit per invested appropriation effort unit of *a* is thus dependent on the current size of the resource, relative to its original size. If *R*_*t*−1_ = *R*_0_, which is the case in the first period of the game, the return is 4 − 1 = 3 units per invested unit of *a*. When *R*_*t*−1_ < *R*_0_, the return will be lower than 3 units. The amount of appropriators’ endowment not used for fishing is reflected by (*E*_*i*_ − *a*_*it*_); players will thus keep the part of the endowment that they did not invest in appropriation as profit for that period.

At the end of the period, the players see how much they invested themselves; their profit from investing; and how much was invested in appropriation of the resource in total as a group. At the beginning of the next period, they also see how much each individual player in their group invested in previous periods.

#### Resource renewal

Just like real natural resources, the resource in the game has a renewal rate. The renewal rate per period is modelled as follows:

$$\begin{array}{c} R_{t}=\text{min}(600,1.25(R_{t-1}-(\frac{R_{t-1}}{R_{0}})\sum_{i=1}^{4}a_{it})) \end{array}$$

Here, 1.25 is the renewal rate of the resource and *R*_*t*_ is the resource size at timepoint *t*. The amount of fish in the lake is thus multiplied by 1.25 after each period. The maximum resource size is *R*_*t*_ = 600; this is the maximum amount of fish in the lake and the resource cannot grow beyond this size. The sum of appropriation effort of all four appropriators is indicated by $\sum_{t=1}^{4}a_{it}$.

#### Overexploitation

The CPR is overexploited—that is, the pool of fish in the lake is smaller than in the previous period—when *R*_*t*_ < *R*_*t*−1_, so when the resource size in timepoint *t* is smaller than in the previous timepoint. This happens if $\sum_{t=1}^{4}a_{it}>120$ because this is the limit of sustainable appropriation based on $R_{0}-\frac{R_{0}}{1.25}$. The CPR is thus overexploited when the four appropriators have invested on average 30 units in appropriation effort per person $\left(600-\frac{600}{1.25}=120\right)$. Investing stops being profitable if $R_{t}=\frac{R_{0}}{4}$, so if the resource size decreased to 25 per cent of the original resource size (*R*_*t*_ = 150), because:

$$\begin{array}{c} U_{it}=(\frac{4}{600}150)a_{it}+(E_{i}-a_{it})=E_{i} \end{array}$$

When this happens, any amount the appropriator invests in appropriation of the resource will result in a return of exactly that amount, and the profit consisting of the return plus the leftover endowment will thus result in *U*_*it*_ = *E*_*i*_. For example, if the appropriator has *E*_*i*_ = 50 and *a*_*it*_ = 50, the return will be 50 + 0 = 50; if the appropriator invests 10 the return will be 10 + 40 = 50 etc. When the resource size drops below 25 per cent of the original size, appropriators make loss by investing in appropriation. Only when the size of the resource increases again will the multiplication of the invested unit of *a* increase and fishing will become relatively more profitable. Graphs visualizing the development of the resource size under different levels of appropriation over time, and the decreasing marginal profits of overexploitation are shown in S2 and S3 Figs respectively.

**Fig 2:**
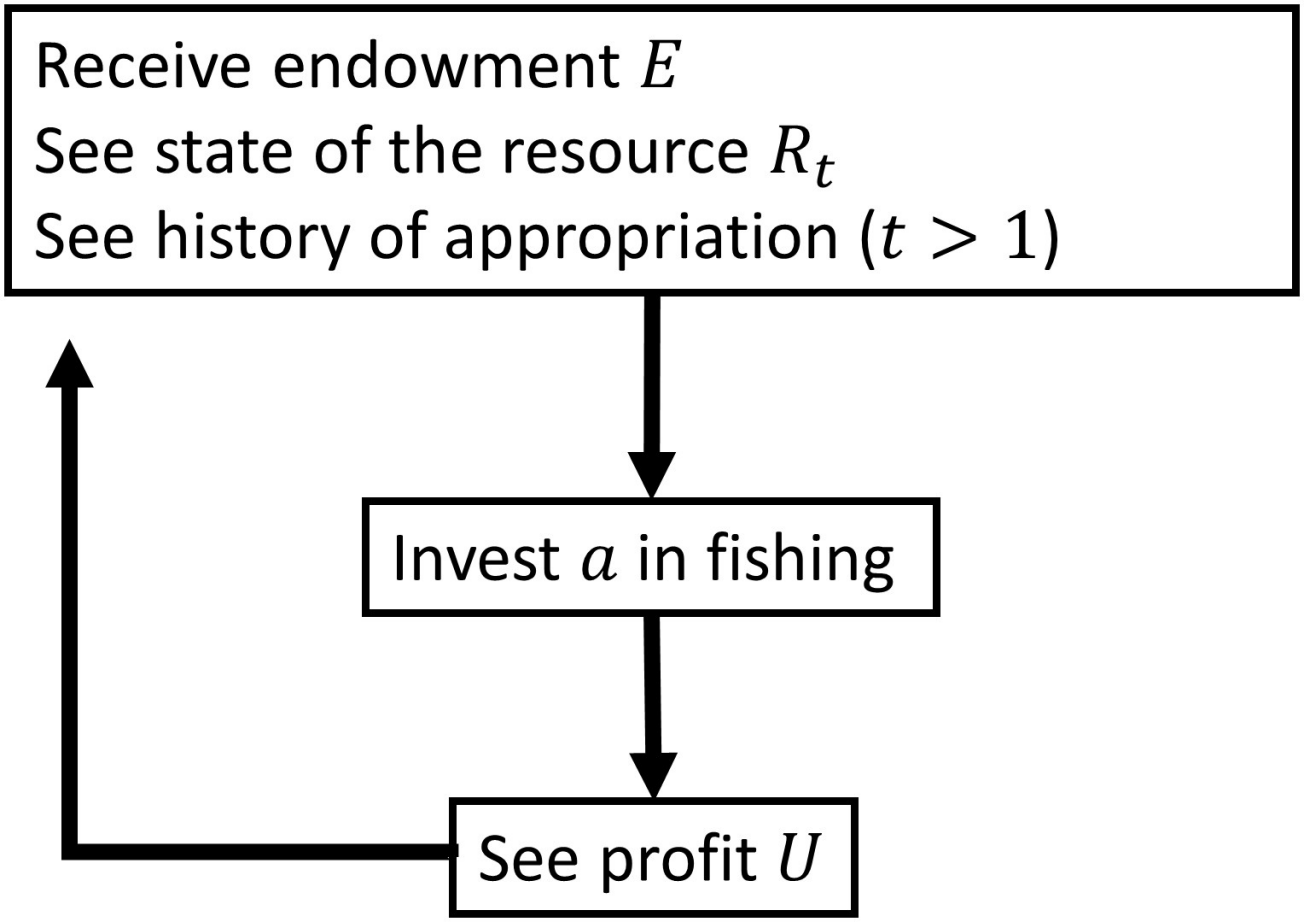
The different stages in a Fishing Game period.

In real life, fishermen may not notice the decline of the resource size within a day’s time. However, having limited time for a laboratory experiment, the game unfolds as if the process of depletion and renewal was sped up. With a total number of 40 periods the experiment covers enough time to capture long-term behaviour. Fig 2 schematically visualises the different stages in a period of the Fishing Game.

#### Trust

Trust is measured at three points in the experimental session: 1) by the one-shot Investment Game with a random other subject at the very beginning of the experimental session, before the MGE and the main game; 2) after the MGE when subjects play the one-shot Investment Game once with an ingroup member and once with an outgroup member; and 3) by asking questions on trust in a post-experimental survey. The one-shot Investment Game with a stranger will be used to measure general trust. Even if this type of trust does not resemble the mutual trust necessary for repeated games (such as the current CPR game) exactly, it is still useful as an indicator of general trustfulness of subjects before they enter the main game.

### Experimental treatments

#### Sociocultural heterogeneity: The MGE

To test the effect of sociocultural heterogeneity on cooperation, an MGE is used to create artificial identities which are based on a trivial criterion [38–40, 67]. Following the approach of amongst others Tajfel, Billig, Bundy and Flament [39], Masella, Meier and Zahn [41] and Aksoy [40, 81], the subjects are shown five paintings by two artists, Paul Klee and Wassily Kandinsky, after which they are asked to express their preference of either picture, resulting in a score of 1 to 5 for Klee-preference. Based on the median preference of the particular experimental session, subjects are assigned to the Klee or Kandinksy group. Tajfel et al. [39], in one of the first published studies using the MGE, divide subjects randomly over groups, regardless of their Klee or Kandinsky preferences. Even random allocation led to subjects trying to maximise ingroup outcomes. However, following Aksoy [40] and specifically using the method of Masella et al. [41], subjects in this study were divided on the actual outcomes of their choices. This approach was taken for several reasons. Firstly, this was done to avoid deception of subjects. Secondly, letting subjects go through a process where they are divided into groups based on a real characteristic, namely preference for Klee or Kandinsky, could add to the feeling of belonging to a group. Thirdly, in the rare case a subject would have a profound preference for either painting, knowing which painter painted which painting, allocating them by chance in the wrong group would decrease the power of the experiment.

After grouping subjects into Klees and Kandinskys, this study follows Aksoy [40] by enhancing group identities with a quiz and an other-other Dictator Game. In the quiz, players have to guess the painter (Klee or Kandinsky) of three paintings. High group performance is profitable: if more than half of the answers of the ingroup are right and/or if the ingroup has more right answers than the other group, players from that group get extra points. The extra points from this part of the experiment will be shown at the end of the CPR game, in order to avoid that low group performance in the quiz influences behaviour in the CPR game [40]. Next, subjects play three periods of a binary other-other Dictator Game, as described by amongst others Aksoy and Weesie [82] and Bilancini, Boncinelli, Capraro, Celadin and Di Paolo [83]. In this game, players face three scenarios in which they have to divide points between a Klee and a Kandinsky player (an ingroup and an outgroup member). In each of the three scenarios, they can opt to divide points equally or unequally between both other players. The purpose of this game is to make players aware of their group membership. Each experimental session, one scenario is randomly picked to be a paid interaction in which the choice that players make influences the amount of points that will be sent to a Klee and a Kandinsky player. Subjects know that any one of the three scenarios will be paid. The three scenarios with the available options in the other-other DG are provided in S4 Text.

An alternative way to induce sociocultural heterogeneity would be to use natural identities based on for instance gender, religion or ethnicity [84, 85]. However, it is not necessarily known to what extent, if at all, a subject identifies with their natural identity. Next to that, it is unpredictable how natural identities will respond to experimental manipulations, and there are many other factors that may vary with natural identity that may influence behaviour [40]. Induced identities on the other hand, are fully controllable and unambiguous, allowing for a bigger confidence that any behavioural differences between subjects are indeed caused by the treatment itself [40]. Even though the groups are based on an artificial criterion, plenty of research shows that the feeling of belonging to a group, no matter on what basis categorisation takes place, is enough to create social identity [39, 40, 59, 67, 86].

It was checked and confirmed that being a Klee or a Kandinsky itself did not significantly affect the outcomes of interest, that is, no underlying behaviour was associated with becoming a Klee or Kandinsky.

#### Economic heterogeneity

Economic heterogeneity is induced by varying the endowment *E*_*i*_ that players receive at the start of each period. Under economic homogeneity, all appropriators receive *E*_*i*_ = 50 to invest in appropriation. Under economic heterogeneity, however, two appropriators receive *E*_*i*_ = 40 and two appropriators receive *E*_*i*_ = 60 (See [13] for a similar operationalisation of economic heterogeneity based on variations in endowment). The total endowment of the group is 200 for all groups in the experiment.

#### Four combinations

The four treatments that are applied in the experiment are shown in Table 2. Subjects know their own endowment and the endowment of others in their group; just as they know their own preference identity (Klee or Kandinsky) and the preference identity of others in their group. They see all this information in a box on the screen every period.

**Table 2:** Overview of treatments.

	Treatment	Operationalisation
EH	Economic heterogeneity	Different endowments
	Sociocultural homogeneity	Same preference identities
SH	Economic homogeneity	Same endowments
	Sociocultural heterogeneity	Different preference identities
EHSH	Economic heterogeneity	Different endowments
	Sociocultural heterogeneity	Different preference identities
NH	Economic homogeneity	Same endowments
	Sociocultural homogeneity	Same preference identities

Randomisation of subjects across groups in the main game was organised as follows. First, a rank order was created with subjects ranked on how many Kandinsky paintings they preferred out of five paintings. To prevent subjects sharing a rank, a random number between 0 and 1 was added to their rank, and the list was ordered again. From this ordered list, the bottom half was declared a Klee, and the top half Kandinsky. Groups were then assembled according to the treatment for that group; homogeneous for the EH and NH treatments (all Klees or Kandinskys) or heterogeneous for the SH and EHSH treatments (two Klees and two Kandinskys). Groups are assigned to treatments manually, depending on the number of subjects in a session and the total number of subjects in each treatment from earlier experiments.

Note that in the EHSH treatment, economic heterogeneity is lined up with sociocultural heterogeneity; that is, two members of the same identity group receive *E*_*i*_ = 60 and the two other members, who are both members of the other identity group, receive *E*_*i*_ = 40. In the EH treatment, two randomly chosen players receive *E*_*i*_ = 40 and the other two receive *E*_*i*_ = 60. This design does not include a treatment where economic and sociocultural heterogeneity are not lined up: economic heterogeneity is only found within and between groups, not both. This was done for two reasons. Firstly, by only varying heterogeneity within and between groups, the experimental design is kept simpler; adding a fifth treatment would result in fewer subjects in each treatment, or the need for a larger sample size to retain statistical power. Secondly, lining up economic and sociocultural heterogeneity enables one to distil whether it is economic or sociocultural heterogeneity that plays a role in certain conflicts between appropriators, such as the conflicts between Japanese and Chinese fishermen between 1920 and 1930 and after World War II: two groups who differed greatly in culture and in fishing assets [87, 88]. However, this does not mean that there are no cases where economic and sociocultural heterogeneity are not lined up. An interesting experiment that varies the overlap between economic and sociocultural heterogeneity is Aksoy [81].

### Analytical strategy

#### Functions

A multilevel regression framework is deployed to test the hypotheses outlined above. For the macro-outcome resource size, a two-level multilevel model will be fitted with period-level outcomes (level 1) and random intercepts for groups (level 2). Individual characteristics will be aggregated to the group level—i.e. the group mean of general trust from the IG, sex, age, game theory experience etc. will be taken. The model is represented in the following function:

$$\begin{array}{cc} y_{tj}=\alpha_{j} & +\sum_{k=1}\beta_{k}x_{jk}\\ & +\sum_{\tau =1}\psi_{\tau }z_{j\tau }+\omega g_{j}\\ & +\phi_{1}t+\phi_{2}t^{2}\\ & +\sum_{\tau =1}\theta_{\tau }\left(z_{j\tau }\times t\right)+\sum_{\tau =1}\lambda_{\tau }\left(z_{j\tau }\times t^{2}\right)+\xi \left(g_{j}\times t\right)\\ & +e_{tj}; \end{array}$$

with $\alpha_{j}∼N\left(\mu^{\alpha },\sigma_{\alpha }^{2}\right)$ and *e*_*tj*_ ∼ *N*(0, *σ*^2^). *α*_*j*_ indicates the intercept for groups. There are *t* periods for *j* groups; *k* control variables *x* with coefficient *β*; *τ* treatments *z* with coefficients *ψ*. *g*_*j*_ represents average general trust as measured by the IG per group *j* with coefficient *ω*. Finally, an interaction of treatments with period and the quadratic term of period with respectively coefficients *θ* and λ, and an interaction of average general trust with period with coefficient *ξ* are included.

For the micro-outcome individual appropriation effort, a three-level multilevel model will be fitted with period-level outcomes (level 1) and random intercepts for individuals (level 2) and groups (level 3), as represented in the following function:

$$\begin{array}{cc} y_{tij}=\Psi_{i}+\alpha_{j} & +\sum_{k=1}\beta_{k}x_{ijk}\\ & +\sum_{\tau =1}\psi_{\tau }z_{ij\tau }+\omega g_{ij}\\ & +\phi_{1}t+\phi_{2}t^{2}\\ & +\sum_{\tau =1}\theta_{\tau }\left(z_{ij\tau }\times t\right)+\sum_{\tau =1}\lambda_{\tau }\left(z_{ij\tau }\times t^{2}\right)+\xi \left(g_{ij}\times t\right)\\ & +e_{tij}; \end{array}$$

with $\Psi_{i}∼N\left(\mu^{\Psi },\sigma_{\Psi }^{2}\right)$, $\alpha_{j}∼N\left(\mu^{\alpha },\sigma_{\alpha }^{2}\right)$ and *e*_*tij*_ ∼ *N*(0, *σ*^2^). Ψ indicates the intercept for individuals, and *α* indicates the intercept for groups. There are *t* periods for *i* individuals in *j* groups; *k* control variables *x* with coefficient *β*; *τ* treatments *z* with coefficient *ψ*. *g*_*ij*_ represents individual general trust as measured by the IG for person *i* in group *j* with coefficient *ω*. An interaction of treatments and period and the quadratic term of period with respectively coefficients *θ* and λ, and an interaction of individual general trust with period with coefficient *ξ* are included.

#### Controls

The macro-model on resource size controls for within-group levels of average general trust as measured by the IG, average age, average number of real-life acquaintances in the experimental session, average game theory experience, percentage of students, percentage of women, and whether a session took place in the Netherlands (1) or not (0). The micro-model on appropriation effort controls for the group means as listed above, plus the individual measures of general IG trust, age, sex (male 0, female 1), number of acquaintances, game theory experience and being a student. All of the controls are present in the models, but only the ones that impact the dependent variable significantly will be reported in the tables.

In addition, the micro-model controls for resource size at period *t* − 1, to tease out how much of the individual behaviour is due to the actual treatment instead of the resource size in the previous period; one can expect a general tendency of individuals to appropriate more when the resource size is bigger, and less when the resource size is smaller. The macro-model on resource size will not control for this, for two reasons. Firstly, resource size is a direct and absolute measure of success at the macro level. Controlling for resource size in the previous period would change the interpretation of the dependent variable into periodic change in resource size, which is unintuitive as a measure of success. Secondly, as change in resource size is potentially affected by the treatment, controlling for resource size in the previous period could create endogeneity, which would complicate the interpretation of treatment effects from the model.

Instead of controlling for lagged resource size, period by treatment interactions are introduced up to a second order polynomial to account for the explicitly dynamic nature of the treatment effects. A critic may hold that such a model specification may be ad-hoc or otherwise mis-specified; the response can be found in S4 and S5 Figs in the supporting information, showing that non-parametric regression splines fitted to the purged residuals using Generalised Additive Models [89, 90] come to similar conclusions regarding the nature of the dynamic functional forms of the treatments.

While it is common in analyses of experimental data to control for endgame effects, this is not the case in the current analyses. Endgame effects are found when participants behave in a purely selfish and profit optimizing way near the end of the game, as they know that the interaction will end and thus there will be no consequences of defection in the future [91, 92]. In the current game, however, there is no clear endgame behaviour visible, as subjects did not know how many periods of the game they would have to play. As González, Güth and Levati [92] show, not informing participants when the experimental interaction ends does not alter behaviour in the game but does reduce the frequency of endgame effects. In addition, even though the experiment consists of many parts and experimental sessions took a significant amount of time, no sign of exhaustion by players is visible—that is, behaviour throughout the game seems constant with the exception of the first fifteen periods. An effect can be expected for these first few periods of the game: subjects may not know the game well enough to understand the consequences of their behaviour until after the first couple of periods. However, in the current game the initial drop of the resource size is not necessarily an artefact of participants misunderstanding the game but rather a behavioural pattern common in CPR games (see for instance [31] and [93]). Thus, as this is a process that is not an unnatural behavioural response to the game but a process that can be expected to be found in any CPR, the models will not control for startgame effects.

## Results

### Descriptive plots

**Fig 3:**
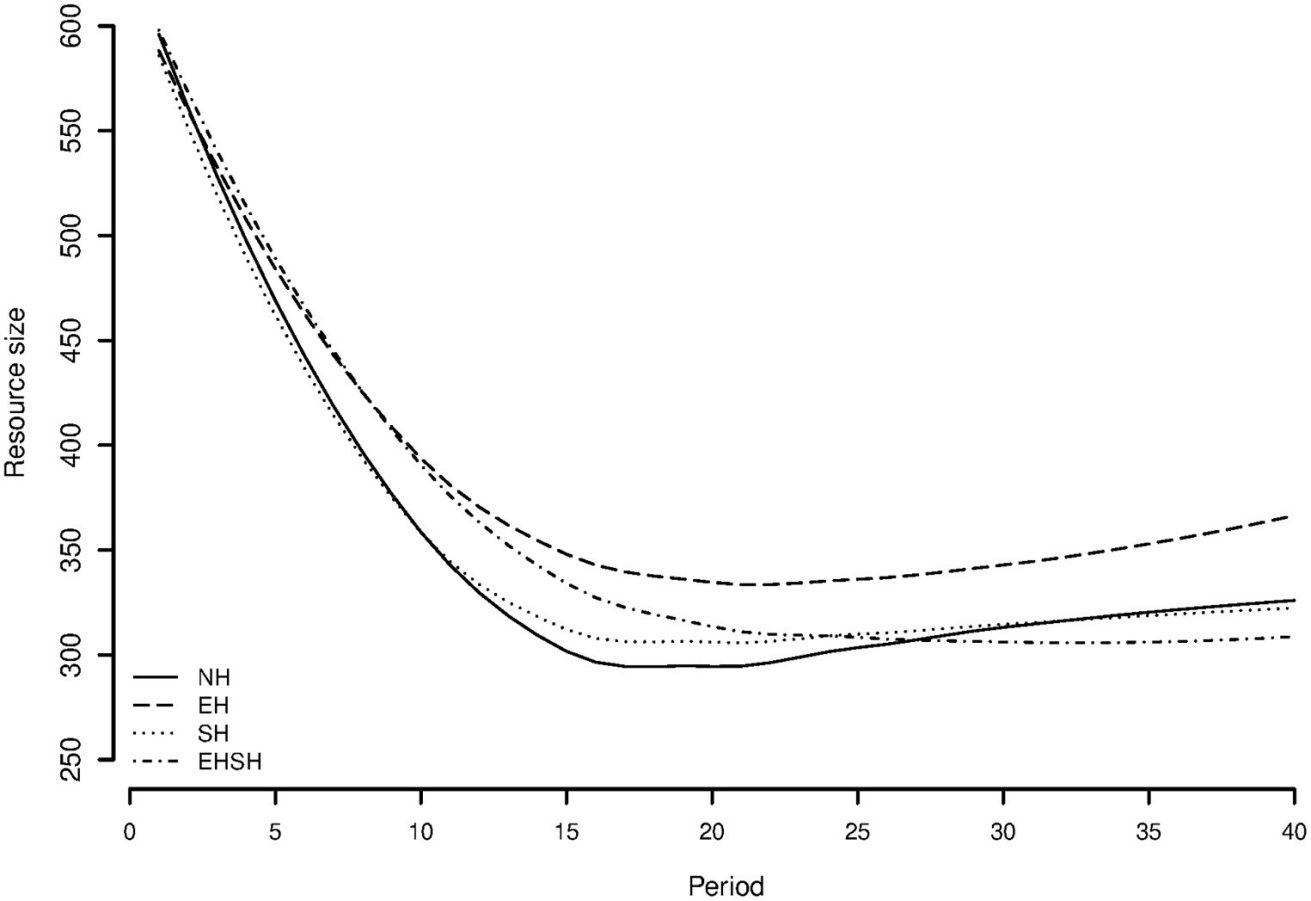
Mean resource size per treatment over time.

Fig 3 shows an interesting difference in resource size over time between the treatments. The first thing that catches the eye is the steep drop of resource size in the first ten to fifteen periods. When the resource is at its fullest, all groups in all treatments seem to overappropriate the resource. This trend is very similar to the trend shown in the CPR game by Janssen, Holahan, Lee and Ostrom [31], under treatments of no communication and punishment and costly punishment. A steep initial drop in resource size is also visible in the first 10 periods of the CPR fishing experiment by Hey, Neugebauer and Sadrieh [93], in a treatment where information resource growth and stock size are available.

Looking at differences between treatments, it stands out that the EH treatment seems to do better—that is, has a higher resource size and thus a higher profit per invested unit of appropriation—throughout the game than the other treatments, including the NH treatment. Strikingly, this only holds for EH under sociocultural homogeneity, and not for the EHSH treatment, in which the resource size keeps decreasing throughout the game and ends up being lower than the NH treatment. Given the curve visible in the graph, treatment effects may vary over time, suggesting an interaction with the quadratic term of time.

**Fig 4:**
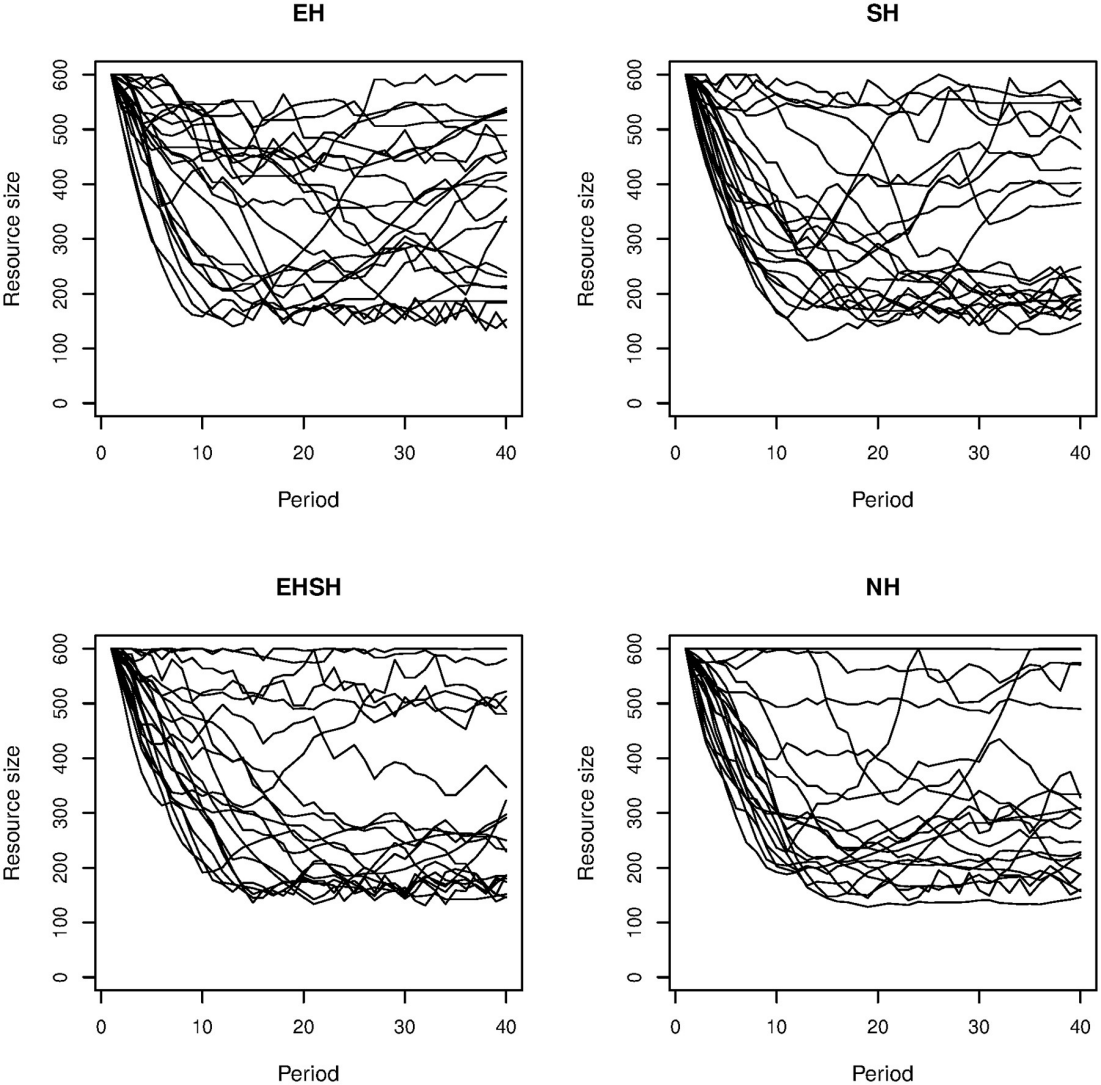
Resource size per group per treatment.

To get a more detailed idea of how groups performed in the various treatments, Fig 4 shows the resource size in each group per treatment. This graph shows that all treatments have well and poorly performing groups. However, it is clearly visible that the EH treatment has the highest concentration of groups that maintain a resource size above 400 throughout the game. The EHSH treatment has the highest concentration of groups that have a resource size below 300 throughout the game.

**Fig 5:**
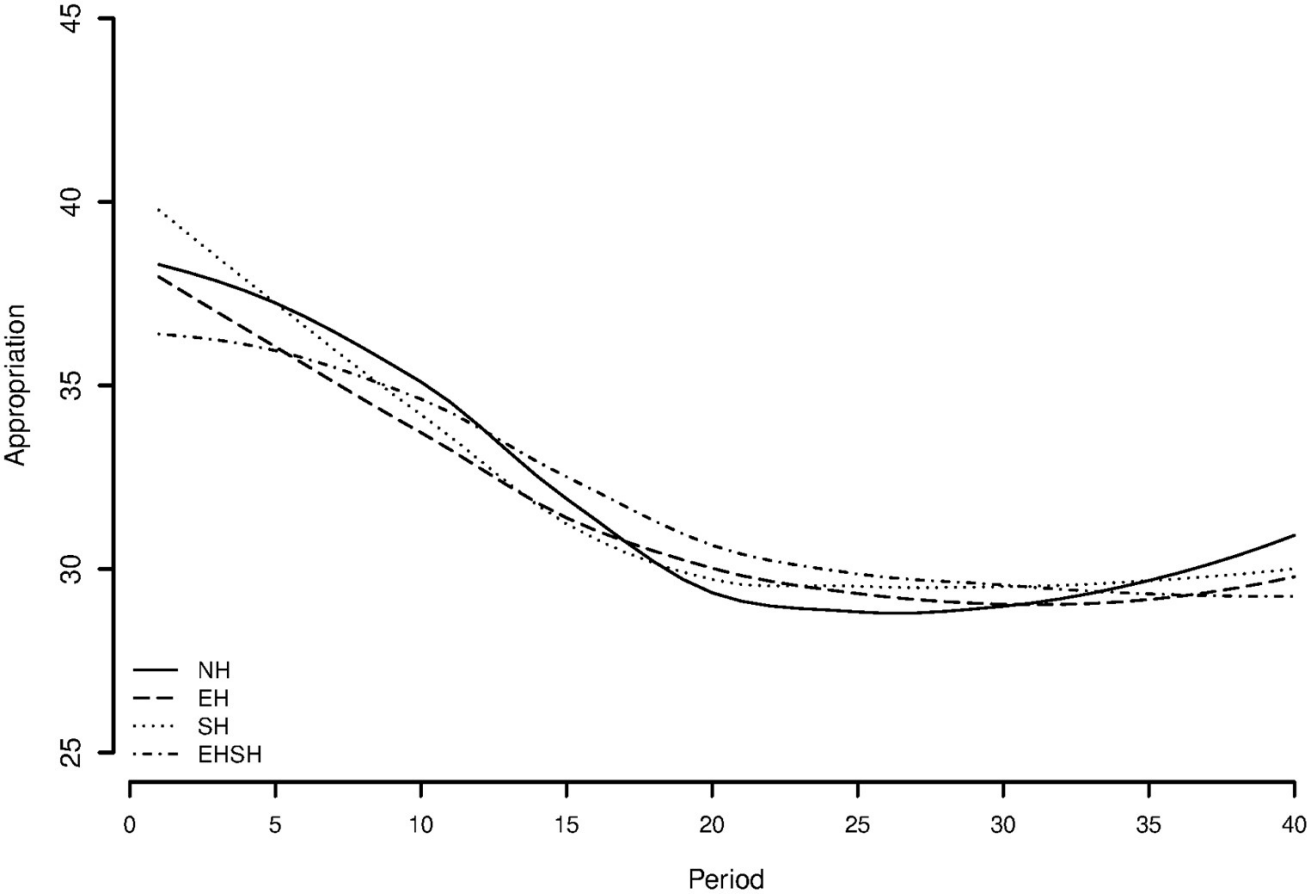
Mean appropriation effort per treatment over time.

Fig 5 shows a behavioural pattern consistent with Fig 3; the EH treatment starts off with and keeps up a decreasing appropriation effort throughout the game. The EHSH treatment decreases too, although with a smaller slope, resulting in overexploitation and a lower resource size as is visible in Fig 3. In all treatments, the appropriation effort stabilizes around 30 units of *a*, which is the cooperative amount to invest in the resource per period per player.

**Fig 6:**
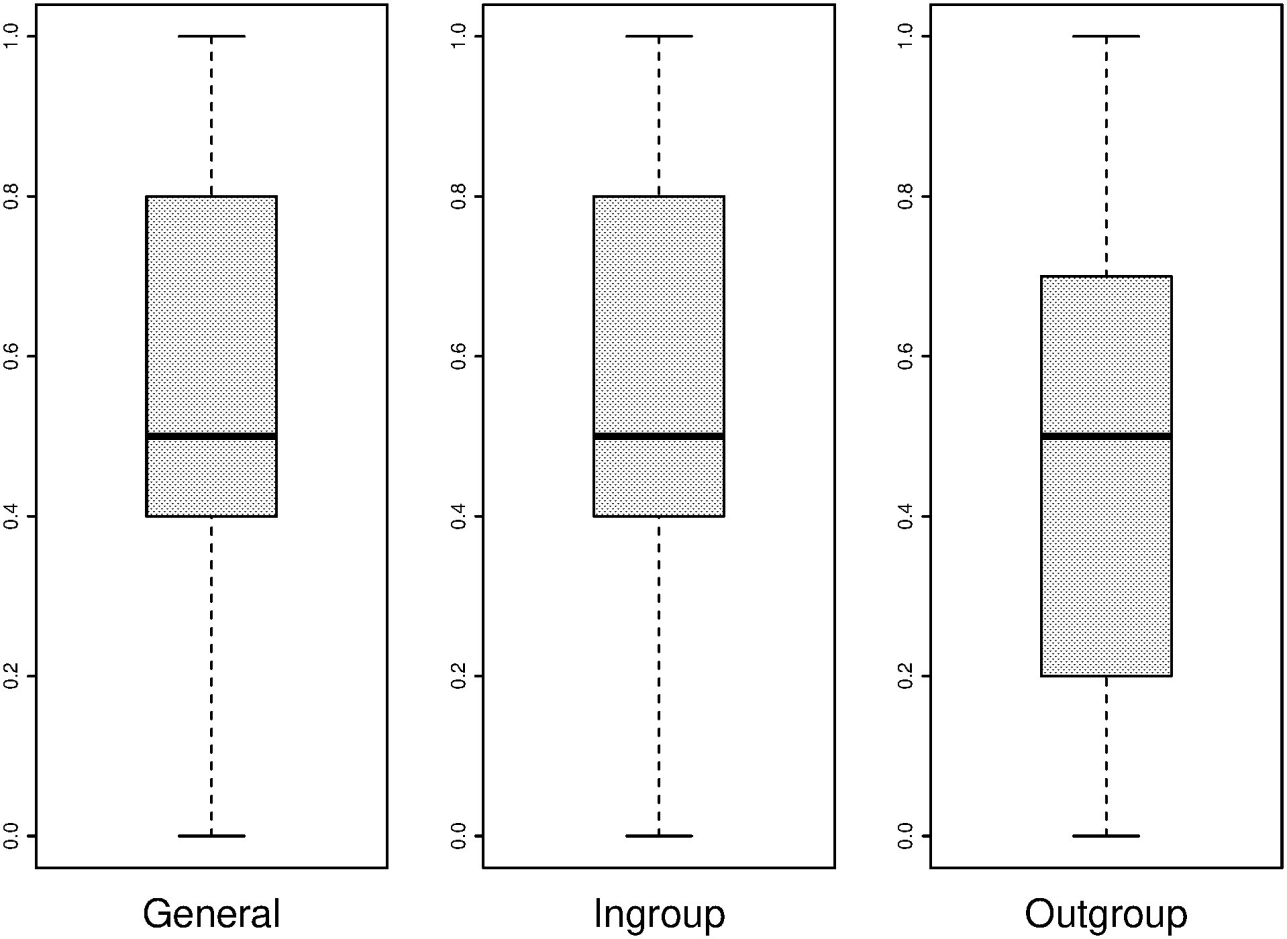
Trust of players in general (left), ingroup (centre) and outgroup (right) players as displayed in the IG.

Fig 6 shows boxplots of general, ingroup and outgroup trust as measured by the general IG, the ingroup IG and the outgroup IG, converted to a measure between 0 and 1. Each section of the box represents a quarter of the observations, and the middle line is the median. The boxplots show that while the trusting behaviour is similar in the general IG and the ingroup IG, it is generally lower for the outgroup IG. The latter illustrates at least partially the implications of the MGE; subjects treat outgroup members differently from ingroup members, even if group membership is based on an artificial criterion.

**Fig 7:**
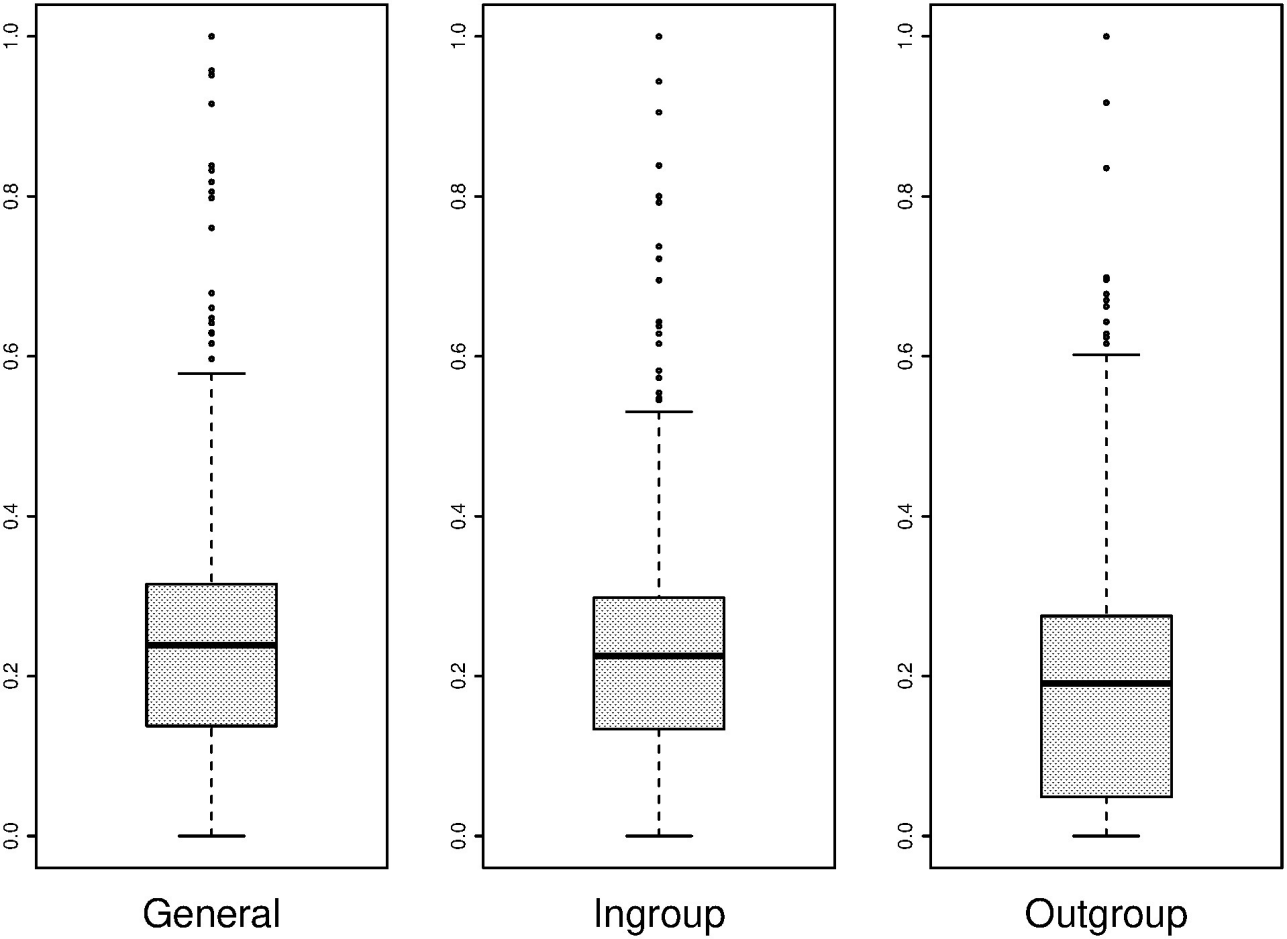
Trustworthiness of players towards general (left), ingroup (centre) and outgroup (right) players as displayed in the IG.

Similar to Fig 6, the boxplots in Fig 7 show a generally lower level of trustworthiness towards outgroup members than ingroup members, while general trustworthiness is similar to the ingroup IG trustworthiness levels.

Based on these descriptive plots, some interesting areas to investigate further include (a) the difference in effects between treatments over time; (b) a potential quadratic relation between treatment effects and time; and (c) the effect of general trust measured with the Investment Game on main-game behaviour of individuals in the different treatments. Results will be analysed separately for the macro- and the micro-level.

### Non-parametric tests

A Kruskal-Wallis test [94] is employed to compare the means of resource size and appropriation effort between the four treatment groups. The test concludes that there is significant evidence that the group means are not equal (*X*^2^ = 27.207; df = 3; p <0.001). Regarding appropriation effort, there is no evidence that the group means are not equal.

To analyse the differences in resource size between specific treatments, Mann-Whitney-Wilcoxon tests are performed for every pair of treatments. The distribution of resource size for EH is found to be significantly different from SH (W = 1233, p <0.001), EHSH (W = 1124, p < 0.001) and NH (W = 1237, p < 0.001). The other treatments do not differ significantly from each other according to the test. However, the trends of resource size and appropriation over time may still differ between treatments, as is apparent from the descriptive plots. Whether this is the case will be investigated using interactions in the multilevel regressions.

### Resource size results

Table 3 shows the two-level multilevel regression on resource size. Model 3 is the model including treatments interacted with the linear and quadratic term of period, trust interacted with period, and all the group characteristic control variables.

**Table 3:** Two-level multilevel regression on resource size with random intercepts for groups.

	(1)	(2)	(3)
EH	21.131	16.152	−1.921
	(36.152)	(35.073)	(35.780)
EH × Period	0.497	0.483	3.064*
	(0.355)	(0.355)	(1.204)
EH × Period^2^			−0.063*
			(0.028)
SH	−1.645	−9.761	−23.581
	(36.532)	(35.365)	(36.081)
SH × Period	0.143	0.135	2.110^+^
	(0.359)	(0.359)	(1.217)
SH × Period^2^			−0.048^+^
			(0.029)
EHSH	35.099	21.953	2.274
	(36.944)	(36.227)	(36.943)
EHSH × Period	−1.236***	−1.236*	1.576
	(0.363)	(0.363)	(1.231)
EHSH × Period^2^			−0.069*
			(0.029)
Period	−4.437***	−5.445***	−23.479***
	(0.260)	(0.513)	(0.954)
Period^2^			0.440***
			(0.021)
General Trust [GT]		106.000	106.000
		(88.263)	(87.787)
GT × Period		1.811*	1.811**
		(0.795)	(0.658)
**Controls**			
Netherlands		88.930**	88.930**
		(30.551)	(30.551)
% Female		94.096^+^	94.096^+^
		(49.836)	(49.836)
Constant	439.853***	471.407**	597.644***
	(26.440)	(165.859)	(165.862)
Observations	3,440	3,440	3,440
Groups	86	86	86
Log Likelihood	−20,323.720	−20,283.180	−19,659.490
Akaike Inf. Crit.	40,667.450	40,602.360	39,362.980
Bayesian Inf. Crit.	40,728.860	40,712.850	39,498.000

Standard errors in parentheses.

^+^p<0.1;

*p<0.05;

**p<0.01;

***p<0.001

Tables produced with *Stargazer* [95]

The main effects of the treatments, to be interpreted as the treatment effect in period *t* = 0, are not significant and are in fact not very meaningful for interpretation on their own.

Model 3 shows a significant positive interaction effect of EH with time (B = 3.064; se = 1.204; p = 0.011), indicating that the slope of EH on resource size is significantly higher each period compared to NH. There is also a negative effect of EH interacted with the quadratic term of period (B = −0.063; se = 0.028; p = 0.027), indicating that the positive slope of EH on resource size by period will flatten out over the course of the game. This is a surprising but interesting result, contradicting hypothesis 1a, stating a negative effect of economic heterogeneity on cooperation (and thus resource size on the macro-level) over time. Instead, taken together with the negative main effect of EH, results show a more complex relation of the treatment effect over time, starting with a negative effect becoming positive, and then flattening out.

There is a marginally significant positive difference in slope of SH on resource size relative to NH over time (B = 2.110; se = 1.217; p = 0.083) and a marginally significant negative interaction effect of SH with the quadratic term of period (B = −0.048; se = 0.029; p = 0.094). Taken together with the negative main effect of SH, this means that relative to NH, SH starts with a lower resource size, but has a larger slope than NH, which flattens out over time.

Model 3 shows a positive interaction of EHSH with period on resource size (B = 1.576; se = 1.231; p = 0.201), but this effect is not significant. What is significant, however, is the interaction of EHSH with the quadratic term of period (B = −0.069; se = 0.029; p = 0.0119). This indicates that the positive effect of EHSH on resource size per period relative to NH will decrease each period, until the slope becomes smaller than the slope of NH—and thus EHSH performs worse—around period 23. The main effect of period is now to be interpreted as the effect of NH over time. The model shows a significantly negative linear effect (B = −23.479; se = 0.954; p < 0.001), and a positive quadratic effect (B = 0.440; se = 0.021; p = < 0.001), indicating that the slope for NH is lower than the other treatments, but that this difference becomes smaller over time.

When changing the reference category in the multilevel model, it shows that the treatments do not differ significantly from each other. That is, EH and EHSH differ significantly from NH, but not from each other or from SH. When taking EHSH as a reference category, the interaction of EH with the linear term of period is not significant (B = 1.489, se = 1.189, p = 0.211). However, note that the p-value is low; this reflects a high probability that subjects in EH performed better in terms of resource size over time than the subjects in EHSH.

**Fig 8:**
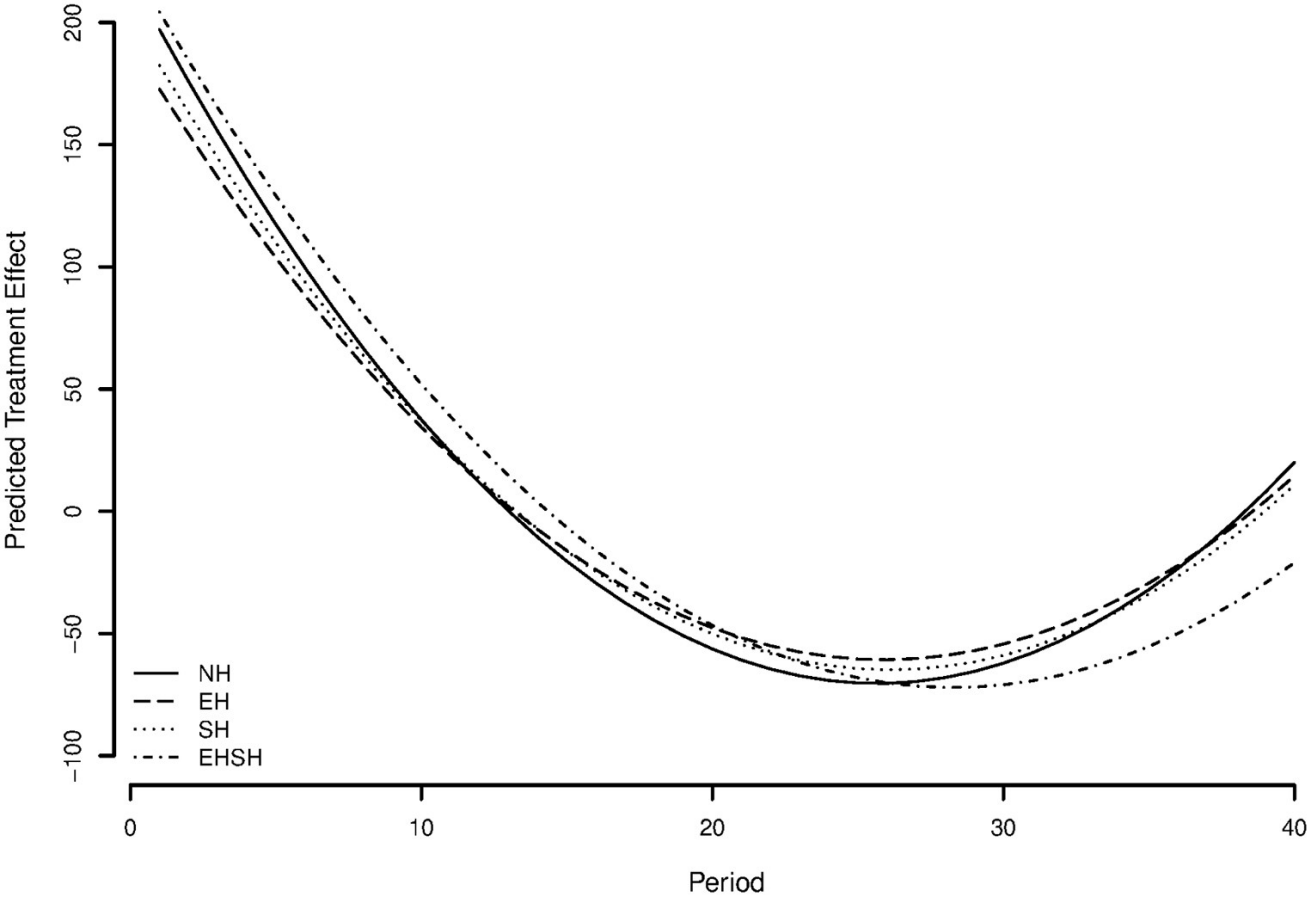
Predicted treatment effects on resource size with multilevel regression coefficients.

To visualise the discussed differences in slopes between treatments, Fig 8 shows a graph of treatment effects fitted on the purged residuals of a model with all control variables. This shows the predicted resource size over time per treatment, while controlling for all relevant control variables.

When put together, these results support hypothesis 1c on the negative effect of economic and sociocultural heterogeneity on cooperation over time. The hypothesis stated specifically a stronger negative effect of the combination of economic and sociocultural heterogeneity than either heterogeneity type separately. While technically true, an effect of economic heterogeneity in the opposite direction and a negligible effect of sociocultural heterogeneity were not anticipated. No evidence was thus found for hypotheses 1a and 1b.

The average level of trust in the group—as measured by taking the group mean of general trust displayed in the Investment Game at the beginning of the experiment—has a significant positive interaction with period (B = 1.811; se = 0.658; p = 0.006) meaning that higher average trust in the group will yield higher resource size each period. This supports hypothesis 3 on the positive effect of trust on cooperation over time. Controlling for the maximum individual level of general trust in the group, the interaction between average level of trust and period is still significant, indicating that it is not just one high trust player in the group that facilitates good outcomes for the group, but that more players with higher trust will lead to better results for the group.

As for the significant control variables, the models show that the subjects in the Netherlands managed to keep up higher levels of the resource size (B = 88.930; se = 30.551; p = 0.005), which is an interesting finding indicating that even though the subject pools from Oxford and Utrecht contain international students and residents of approximately the same age range, the location of the experimental sessions (or perhaps the country of residence of subjects) matters. A marginally significant and positive effect is found for a higher percentage of females in the group (B = 94.096; se = 49.096; p = 0.063).

### Appropriation effort results

Table 4 shows the three-level multilevel regression on the micro-level variable appropriation effort. None of the models show a significant main effect of the treatments, but as these effects are interpreted as treatment effects at period *t* = 0, these coefficients are not very meaningful.

**Table 4:** Three-level multilevel regression on appropriation effort with random intercepts for subjects and groups.

	(1)	(2)	(3)
EH	−0.910	−1.222	−2.103
	(1.287)	(1.430)	(1.620)
EH × Period	0.023	0.017	0.148
	(0.025)	(0.026)	(0.112)
EH × Period^2^			−0.003
			(0.003)
SH	−0.086	0.141	0.368
	(1.300)	(1.450)	(1.644)
SH × Period	0.004	0.003	−0.033
	(0.025)	(0.027)	(0.114)
SH × Period^2^			0.001
			(0.003)
EHSH	−0.548	−0.700	−2.517
	(1.315)	(1.475)	(1.668)
EHSH × Period	0.024	0.036	0.290*
	(0.026)	(0.027)	(0.114)
EHSH × Period^2^			−0.006*
			(0.003)
Period	−0.242***	−0.095***	−0.516***
	(0.018)	(0.026)	(0.090)
Period^2^			0.009***
			(0.002)
General Trust [GT]		−1.689	−1.728
		(1.846)	(1.807)
GT × Period		−0.095**	−0.092**
		(0.030)	(0.030)
**Controls**			
Resource Size t-1		0.017***	0.012***
		(0.001)	(0.001)
Sum Appropriation Others t-1		0.042***	0.036***
		(0.004)	(0.004)
Netherlands		−2.085^+^	−1.625
		(1.179)	(1.153)
Female		−2.006^+^	−1.992^+^
		(1.132)	(1.106)
Constant	36.914***	24.938***	30.795**
	(0.941)	(6.503)	(6.421)
Observations	13,760	13,299	13,299
Subjects	344	341	341
Groups	86	86	86
Log Likelihood	−54,226.350	−52,376.450	−52,370.950
Akaike Inf. Crit.	108,474.700	104,804.900	104,801.900
Bayesian Inf. Crit.	108,557.500	104,999.700	105,026.700

Standard errors in parentheses.

^+^p<0.1;

*p<0.05;

**p<0.01;

***p<0.001

Tables produced with *Stargazer* [95]

Model 3 is the complete model including treatments interacted with the linear and quadratic term of period, trust interacted with period, and control variables. The model shows a significant positive effect of EHSH on appropriation per period relative to NH (B = 0.290; se = 0.114; p = 0.011) but a negative effect when interacted with the quadratic term of period (B = −0.006; se = 0.003; p = 0.020). Taken together with the negative main effect (B = −2.517; se = 1.668), this suggests that the difference in slopes between EHSH and NH on appropriation increases and finally flattens out. Subjects in EHSH thus appropriate more for a large part of the game, until appropriation becomes more similar to NH.

No significant effects of the other treatments are visible, but it is worth noting that for EH the p-values of the main effect (p = 0.198), interaction with linear period (p = 0.186) and interaction with the quadratic term of period (p = 0.238) are close to 0.2, which reflects a non-significant but relatively high probability that subjects in EH treatment behaved more cooperatively over time than subjects in NH, as was found in the macro-model. The main effect of period is now to be interpreted as the effect of NH over time. The model shows a negative slope over time (B = −0.516; se = 0.09; p < 0.001) which increases each period (B = 0.009; se = 0.002; p < 0.001).

**Fig 9:**
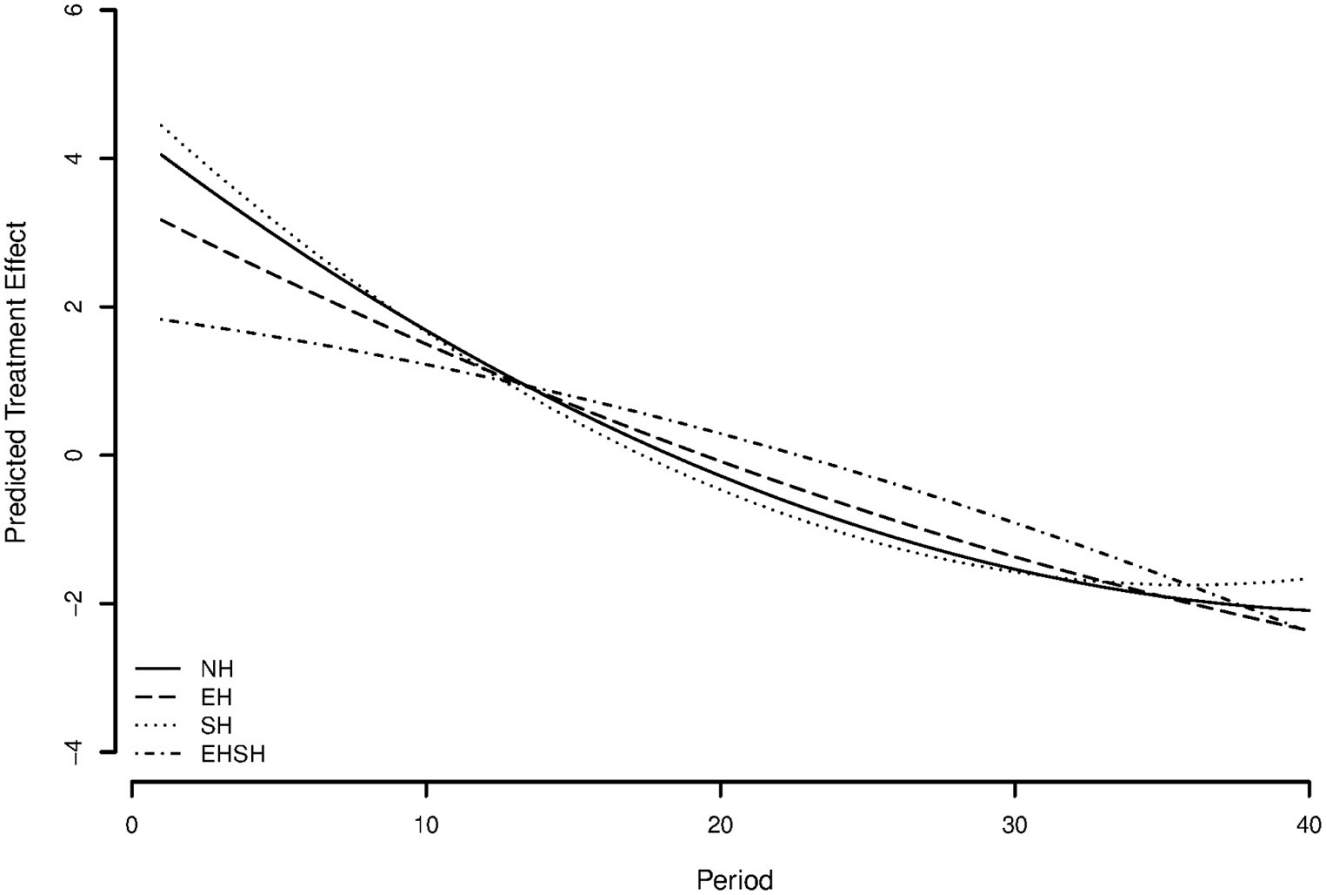
Predicted treatment effects on appropriation effort using multilevel regression coefficients.

To give a visualisation of the differences in slopes between treatments, Fig 9 shows a graph of treatment effects fitted on the residuals of a model with all control variables. This shows the predicted resource size over time per treatment, while controlling for all relevant control variables. The results provide modest evidence in the opposite direction of hypothesis 1a, suggesting for the individual level a positive instead of a negative effect of economic heterogeneity under sociocultural homogeneity on cooperation over time. A surprising but interesting result that will be reflected on later.

It is worth noting the possibility that one would need more statistical power to detect significant differences on the micro-level. However, even small changes in behaviour on the micro-level can lead to big effects at the macro-level. The lack of significant findings on the micro-level thus does not mean that the effects do not play a role, in particular because the differences in behaviour between treatments found on the micro-level in Table 4 do support the differences that were found in Table 3 on the macro-level.

Regarding trust from the IG, model 3 shows a significant negative interaction effect of trust with time on appropriation effort (B = −0.092; se = 0.03; p = 0.002). This suggests that higher levels of general trust result in lower levels of individual appropriation every period and thus higher levels of individual cooperation over time—after all, a lower appropriation effort yields higher resource size and higher returns for every player in the group. This supports hypothesis 3, stating the positive effect of trust on cooperation over time.

Regarding significant control variables, resource size in *t* − 1 (B = 0.012; se = 0.001; p < 0.001), and the sum of appropriation of other players in *t* − 1 (B = 0.036; se = 0.004; p < 0.001) have positive effects on appropriation effort. Female subjects appropriate marginally less (B = −1.992; se = 1.106; p = 0.073). Model 2 shows that Dutch subjects appropriate marginally less (B = −2.006; se = 1.179; p = 0.081), but this effect disappears in model 3.

### Post-experimental trust results

**Fig 10:**
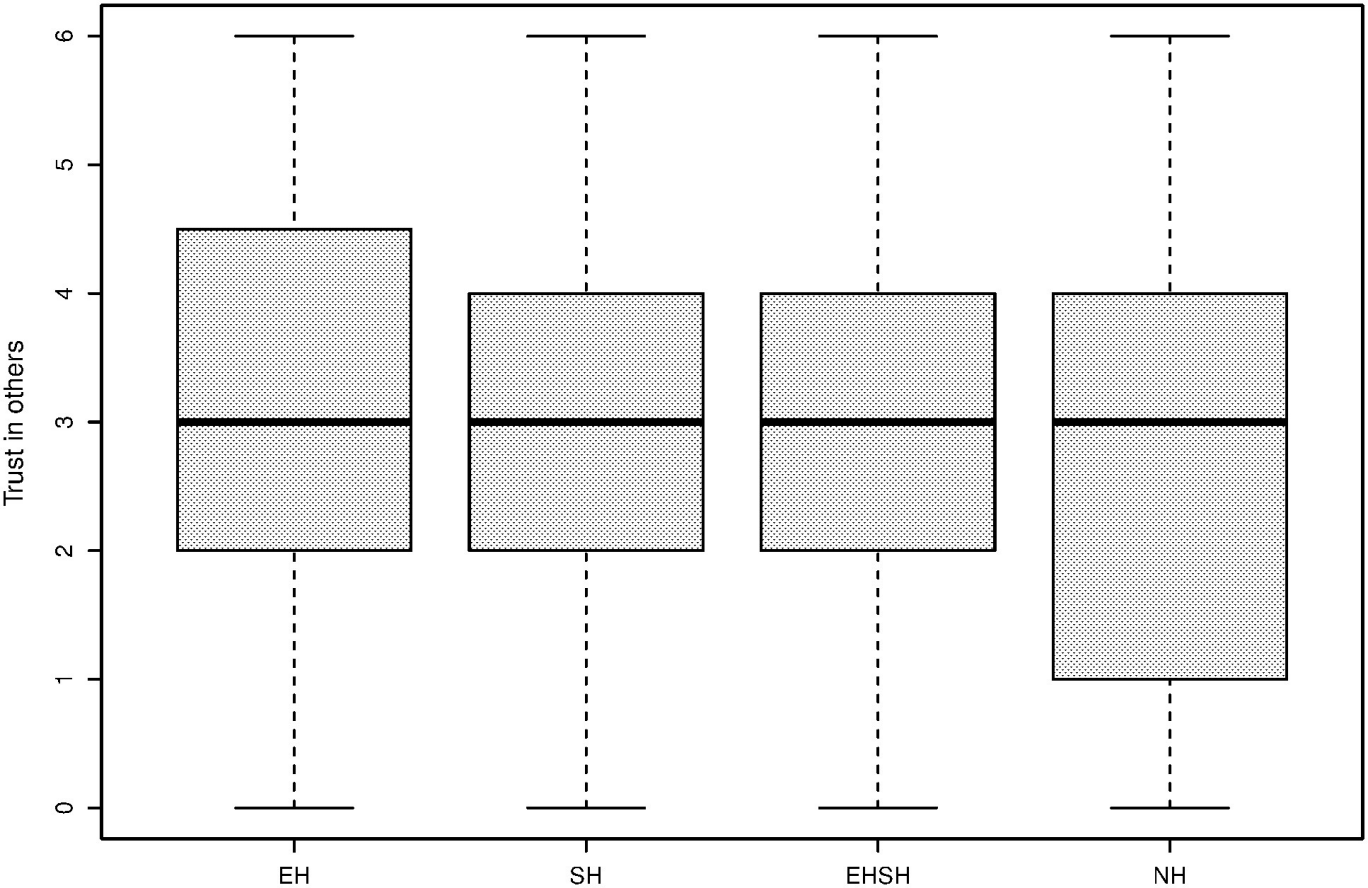
Trust in other players by treatment.

**Fig 11:**
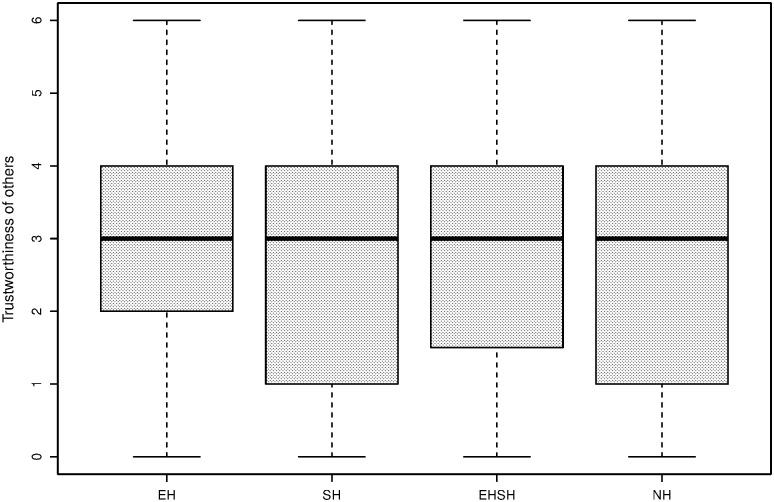
Subjective trustworthiness other players by treatment.

First of all, Fig 10 shows a descriptive plot of trust as measured in the post-experimental survey with the statement “I trusted the other players in my group”, referring to the Fishing Game, hereafter called trust in other players. The variable is measured on a 7-point Likert scale ranging from ‘completely disagree’ (0) to ‘completely agree’ (6). It shows that in especially the economic heterogeneity group, the trust question is answered more positively by a higher percentage of subjects, and for the homogeneity treatment this is lower. Fig 11 shows a descriptive plot of a statement from the post-experimental survey stating “The other players in my group were trustworthy”, hereafter called subjective trustworthiness of other players, and measured on the same 7-point Likert scale. It shows slightly lower scores for the sociocultural heterogeneity treatment and the homogeneity treatment.

An ordinal logistic regression on the two mentioned trust questions from the post-experimental survey can be found in S5 Text. No statistical evidence is found for hypotheses 2a, 2b or 2c on the negative effect of heterogeneity on trust. Instead it seems that the final individual profit at the end of the experiment has a strong positive influence on how the survey questions on trust were answered.

### Revisiting expectations

Table 3 shows that economic heterogeneity affects collective action and resource size in a positive way compared to homogeneity. This is in stark contrast with hypothesis 1a, stating a negative effect of economic heterogeneity. As not only a non-effect but a significant effect in the opposite direction in the macro-model, it is worth revisiting the literature for possible explanations.

#### The Olson-effect

Despite the majority of research suggesting a negative effect of heterogeneity on cooperation, the positive effect of heterogeneity is theorized by the economist Mancur Olson in his book *The logic of collective action: public goods and the theory of groups*. [9] describing what is known as the “Olson-effect”:

“In smaller groups marked by considerable degrees of inequality—that is, in groups of members of unequal “size” or extent of interest in the collective good—there is the greatest likelihood that a collective good will be provided; for the greater the interest in the collective good of any single member, the greater the likelihood that that member will get such a significant proportion of the total benefit from the collective good that he will gain from seeing that the good is provided, even if he has to pay all of the cost himself” (p. 34).

Even though Olson does not directly mention a positive effect of economic heterogeneity on cooperation, he does describe a theoretical mechanism of the rich bearing the costs of cooperation for the poor by overinvesting in cooperation. In the context of the current CPR experiment, the group was small enough so that subjects with higher endowments may have invested less than they could have, to provide space for the two other subjects with lower endowments to invest in the resource for profit. The cost for not investing in the resource, and thus not receiving profit from appropriation, is lower for the higher endowed subjects, as everyone can keep the endowment that was not invested, which is higher for them to begin with.

**Fig 12:**
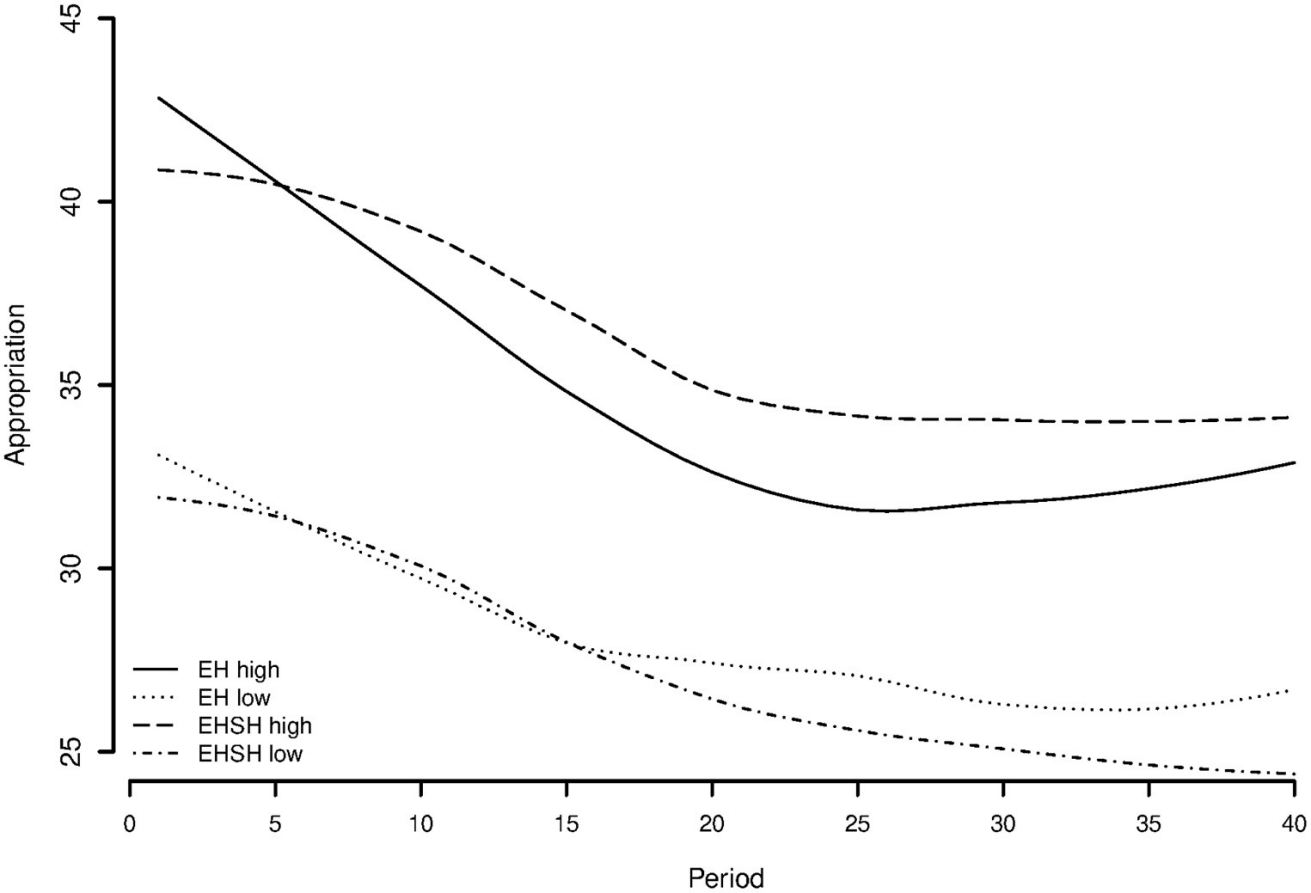
Appropriation of higher (*E* = 60) and lower (*E* = 40) endowed players in economic heterogeneity [EH] and economic and sociocultural heterogeneity [EHSH] treatment (smoothed line).

To explore the possibilities of the Olson-effect in the current experiment, Fig 12 shows a plot of the appropriating behaviour of the high and low endowed subjects in the EH and EHSH treatments. It shows that in the EH treatment, the investments in appropriation for the higher endowed subjects are lower than the investments from higher endowed subjects in the EHSH treatment. For both treatments, the lower endowed subjects have on average about the same appropriation over time, with the ones in EH higher from the 16th period on.

**Fig 13:**
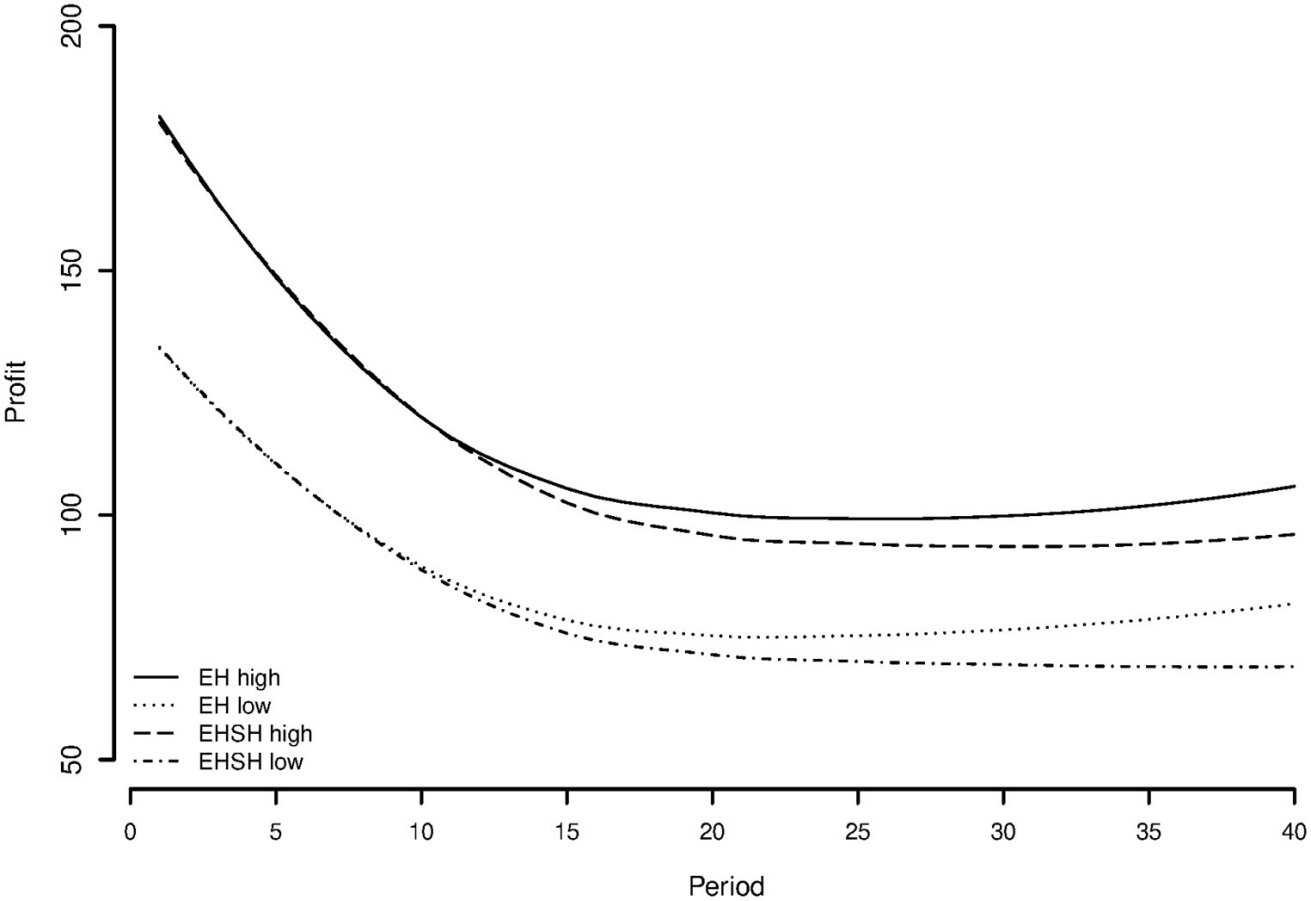
Profit of higher (*E* = 60) and lower (*E* = 40) endowed players in economic heterogeneity [EH] and economic and sociocultural heterogeneity [EHSH] treatment (smoothed line).

Fig 13 shows that the lower endowed subjects in the EH treatment can make more profit than the lower endowed subjects in the EHSH treatment, from about period 10, while higher endowed subjects in EH still make more profit than higher endowed subjects in EHSH. It is still the case that the higher endowed in both treatments overappropriate on average, but an unpaired t-test points out that the higher endowed appropriate less in the EH (M = 34.830, SD = 17.116) than in the EHSH (M = 36.390, SD = 17.922) treatment (t (3518) = −2.642, p = 0.004). Taken together these results provide evidence for the Olson-effect in EH, but not in EHSH.

#### Ingroup favouritism and conditional reciprocity

A closer look can be taken at the effect of the MGE on in-game behaviour. Even though trust itself may not play a role in levels of cooperation, the difference in outcomes of the IG with ingroup and outgroup members could indicate why less cooperative behaviour takes place when economic heterogeneity is combined with sociocultural heterogeneity. Consistent with the descriptive plots of Figs 6 and 7, a paired samples t-test comparing the average trust in the general IG (i.e. playing with a random other player before the MGE) with average trust in the outgroup IG (i.e. playing with an outgroup member after the MGE) shows that there is a significant difference in average trust between a general (M = 0.559, SD = 0.310) and an outgroup (M = 0.455, SD = 0.333) interaction (t (343) = −8.023, p < 0.001). The average trust in the ingroup (M = 0.573. SD = 0.320) and outgroup (M = 0.455, SD = 0.333) IG interaction is also significant (t (343) = 10.010, p < 0.001). These results show that even with a division that is as artificial as painting preferences of painters in the same art discipline, group identities are strong enough to behave differently in different group compositions of ingroup and outgroup members. In their meta-study, Balliet, Wu and De Dreu [96] support the finding that people are more cooperative with ingroup, compared to outgroup, members.

The current results regarding trust indicate derogation towards the outgroup rather than favouritism towards the ingroup, since the difference in behaviour towards a general other and an ingroup member is not significant. In a mixed-design experiment using Dictator Games, Bilancini et al. [83] show that ingroup favouritism is stronger when group are based on moral preferences rather than non-moral preferences. This finding provides another reason for future research to vary the operationalisation of sociocultural heterogeneity.

Another potential explanation for cooperative behaviour in EH is conditional reciprocity. Numerous studies, both experimental and in the field, have shown that people are conditional cooperators: they cooperate when others cooperate as well (see amongst others [97–102]). In the IG results of the current study, conditional reciprocity is visible: controlling for individual characteristics and location of the experiment, player 2 in the general IG sends a significantly higher percentage of their total points back to player 1, the higher the percentage of the points sent by player 1 of their endowment (B = 0.570, se = 0.091, p = < 0.001). Similarly, the significant positive effect of lagged sum of appropriation of others in Table 4 means that a lower appropriation of other players results in a lower appropriation of the player, which can be thought of as a form of reciprocal cooperation. This behaviour can also be related to informational effects; a phenomenon that describes how individuals will better comply with norms or rules if they see a greater number of other individuals complying as well [103].

However, none of the behavioural theories above explain the difference between EH and EHSH behaviour on their own. The answer may lie in a cross between ingroup favouritism, conditional reciprocity and the Olson-effect.

## Discussion

The aim of this paper is to study the impact of economic and sociocultural heterogeneity and the coincidence of the two, through trust on cooperation on the micro- and macro-level in common-pool resources. Using a CPR game in the laboratory allowed for the effects of economic and sociocultural heterogeneity and trust to be disentangled and enabled establishing the causal direction of effects. Existing literature predominantly suggests negative effects of heterogeneity on trust and on cooperation, and positive effects of trust on societal outcomes.

The results show that under the coincidence of economic and sociocultural heterogeneity, groups struggle to converge to a sustainable appropriation of the common-pool resource over time. Surprisingly, the economic heterogeneity treatment is the first to converge to cooperative levels of appropriation. It manages to hold the highest resource size over the 40 periods of the game and is the most successful of all four treatment groups, including the homogeneous control group, over time. A striking conclusion here is thus that it is the presence or absence of sociocultural heterogeneity in common-pool resource settings that makes economic heterogeneity perform respectively worse or better than full homogeneity. The results contribute to the current literature by providing a possible explanation for the emergence of aversion to economic inequality, which may not have a natural origin per se, but could have a sociocultural origin instead. Sociocultural heterogeneity under economic homogeneity, however, differs only marginally from full homogeneity on the macro-level. Regarding trust, it is found that over time, higher average general trust within a group increases resource size, and individual general trust decreases appropriation effort—trust thus increases cooperation on both the micro- and macro-level. In addition, the post-MGE Investment Game results showed that subjects acted more trusting towards ingroup members than towards outgroup members, and less trusting towards outgroup members than to a random other person in the pre-MGE Investment Game. However, no evidence for the mediating role of trust between heterogeneity and cooperation in CPRs was found.

The results support literature suggesting positive effects of economic heterogeneity [9] or U-shaped relations between CPR performance and economic inequality (see [47, 104]). Olson [9] suggests that economic heterogeneity may have a positive effect on cooperation, provided that the rich act as catalysts for cooperation by bearing the cost of collective action just a little more than the poor. It is shown that subjects with high endowments in the economic heterogeneity treatment invest less on average than subjects with a high endowment in the combination treatment. These results are not unthinkable if one places them in the context of common-pool resources: fishermen who come from the same sociocultural background, speaking the same language and with a similar view on the resource, could be more likely to cooperate with each other, despite economic heterogeneity, than with an unfamiliar actor—such as a large fishing company with a big fleet—that is unlike them on both sociocultural background and economic means. The key here could be a conflict of interests arising at the moment that two groups are unequal in economic means to appropriate a resource without a sociocultural bond to bridge that inequality gap. However, as Bazzi et al. [3] argue, the emergence of coordination depends on whether heterogeneity is shaped by many smaller groups, or a few bigger groups; smaller groups are more prone to find common ground, whereas bigger groups are more prone to ingroup antagonism. The results underline the importance of understanding the influence of various types of heterogeneity, and their interaction, on common-pool resource outcomes.

Some critical comments can be made about this study. A well-known criticism of laboratory experiments using mainly students as their subjects is that they are not representative of situations in the real world, while the results are sometimes presented as real-world outcomes. However, if the aim of the research is to investigate relationships between human behaviour and social, biological or economic contextual variables, experiments are a good way of doing so, regardless of the subject pool [36, 105–108]. To point out causality and to show the effect of a treatment the only assumption necessary is appropriate randomisation, which the laboratory setup provides [109]. In addition, a survey-experiment conducted with a representative sample of a city’s population by Exadaktylos, Espín and Brañas-Garza [110] points out that students are indeed appropriate subjects to study human behaviour with laboratory experiments. The external validity of experiments can be secured as long as the environment under which the results are generated capture essential characteristics of the real-world version of the phenomenon that is being researched [111]. In the current paper, a CPR experiment was conducted which contained key aspects of the way CPRs, and in particular fisheries, work. A first step improvement on this study could be made by using real forms of identity, as real-life heterogeneous communities using CPRs also have to deal with real-life sentiments towards real ingroup and outgroup members. For instance, gender or nationality of subjects could be used to operationalise sociocultural heterogeneity. This could also serve as an extra test for and comparison with using the MGE to investigate whether subjects behave differently in CPR games if they are grouped by a natural rather than a more artificial characteristic. Lastly, future research could investigate whether the group size of different preference or identity groups influences the outcome of the game, as Bazzi et al. [3] suggest.

For future CPR experiments it would also be interesting to add communication and/or punishment between players (see for instance Janssen et al. [31]) or to add a type of risk to overexploitation that is separate from decreasing income (see for instance Bednarik, Linnerooth-Bayer, Magnuszewski and Dieckmann [112] where overharvesting trees increases the chance of flood damage). Adding elements like this can change behaviour drastically, and can create a more realistic setting. A bolder improvement on this study could be made by setting up an artefactual field experiment, better known as a lab-in-field experiment: a controlled environment where artefactual games (such as the Investment Game or the Fishing Game) are played, but with a subject pool that is more like the population of interest. In the current context, this could be a group of actual fishermen.

With the use of detailed and heavily contextualised games, comes a more detailed—and thus limited—interpretation of the results; the results of this study are valuable for common-pool resources, and especially resources with structures similar to fishing grounds. With the information about the resource size (fish stock) available for players to see at the beginning of every new period, the results may not hold in situations where the total allowable catch cannot be correctly measured because the fish population dynamics is unknown [93]. In addition, whereas players could see the history of other players’ actions, this may not always be the case (see for instance Lacomba, Lagos and Perote [113]). Future experiments could vary the amount and accuracy of information about resource growth and stock size, or information on other players’ actions, as it is found to influence players’ appropriation behaviour [93, 113, 114]. Findings may be different for other resource types, such as irrigation systems, where resource renewal is partially dependent on the weather, appropriation is sequential, and farmers already know if someone took more water than allowed before they make their own decision of how much water to take.

The level of detail in the game is necessary to fully understand the different mechanisms at work in different CPRs; games that are too generalised will not provide directed results. However, hypotheses were not formulated based on resource-specific characteristics, so it may well be that the found results would apply to a wider range of CPRs. The flexibility for researchers to adjust a game to represent any type of CPR is a very valuable asset of experimental research, and one that should be deployed more often in social science.

## Conclusion

As far as one should base policy advice on laboratory results alone, the main recommendation is perhaps that management of CPRs cannot be a ‘one size fits all’ solution, but instead that sustainable cooperation in CPRs can be achieved under a flexible management that is adapted to the level and the type(s) of heterogeneity found in a CPR user community. Laboratory experiments are important to tease out the mechanisms at work in social phenomenons. The mechanisms for which this paper provide evidence should be further studied in real-life contexts, first through lab-in-field experiments, and then via large-scale randomised control trials, to ensure they are externally valid instruments of policy.

Understanding cooperative behaviour and trust under different conditions of heterogeneity is a core question within social sciences. The application of this question to common-pool resource situations is vital especially in a time of increasing depletion of these resources, manifested in overfishing, deforestation and unsustainable use of fresh water. It is crucial to understand the behaviour of humans in these contexts, to prevent overexploitation of resources and to promote cooperation between different actors involved. In addition, the growing number of modern commons such as citizen initiatives for food, green energy and infrastructure provide new incentives to investigate human behaviour in and around CPRs [30, 42, 43]. The investigation of relations between heterogeneity, trust and cooperation in common-pool resources is not only important to advance insights within the social sciences, but also demonstrates the importance of social science research for present-day problems.

## Supporting information

S1 Text General instructions. The general experiment instructions that subjects receive upon entering the lab.
(PDF)

S2 Text Specific instructions. Treatment-specific instructions for subjects in the different treatments. Treatments are indicated on the top left of the page with an ‘E’ for economic heterogeneity, ‘S’ for sociocultural heterogeneity, ‘B’ for economic and sociocultural heterogeneity (‘Both’) and ‘N’ for no heterogeneity (the homogeneous control group).
(PDF)

S3 Text The Investment Game. A description of all the characteristics of the Investment Game.
(PDF)

S4 Text Table: Other-other Dictator Game details. A table that shows the two options that subjects faced in each of the three scenarios in the other-other DG.
(PDF)

S5 Text Post-experimental trust models. Ordinal logistic regression on two survey questions on trust in and trustworthiness of other players.
(PDF)

S1 Fig The Investment Game. A graphic representation of the Investment Game as played in the current experiment.
(TIF)

S2 Fig Figure: Resource size under different group behaviour. A graph that shows how resource size develops over time under different appropriation behaviour: from a summed appropriation of 120 to 200 in increments of 10.
(TIFF)

S3 Fig Figure: Cumulative profit under different group behaviour. A graph that shows how cumulative profit of groups develops over time under different appropriation behaviour: from a summed appropriation of 120 to 200 in increments of 10.
(TIFF)

S4 Fig GAM with splines for resource size. A graph to compare predicted treatment effects between multilevel [ML] models and generalised additive model [GAM] models, to check whether the interaction effects of the treatments with the linear and quadratic term of period as specified in the multilevel models fit the natural trend of the data. The time variant variables (period and the interactions of treatments with periods) are specified as smooth terms, which are based on low rank version of splines.
(TIFF)

S5 Fig GAM with splines for appropriation effort. A graph to compare predicted treatment effects between multilevel [ML] models and generalised additive model [GAM] models, to check whether the interaction effects of the treatments with the linear and quadratic term of period as specified in the multilevel models fit the natural trend of the data. The time variant variables (period and the interactions of treatments with periods) are specified as smooth terms, which are based on low rank version of splines.
(TIFF)
