# Supplementary material for: Playing nice in the sandbox: On the role of heterogeneity, trust and cooperation in common-pool resources
Source: PLoS One. 2020 Aug 28;15(8):e0237870. doi: 10.1371/journal.pone.0237870 (PMC7454994; doi:10.1371/journal.pone.0237870)

- Instructions -

Welcome to this experiment and thank you for coming. Please read the following instructions carefully. The instructions will state everything you need to know in order to participate in the experiment. If you have any questions, please raise your hand.

- Earning money -

You can earn money by means of earning points during the experiment. The number of points that you earn depends on your own choices. At the end of the experiment, the total number of points that you earn during the experiment will be exchanged at the rate of:

500 points = 1 GBP

The money you earn will be paid out in cash at the end of the experiment without other players being able to see how much you earned. During the experiment you are not allowed to communicate with other players. Please turn off your mobile phone and put it in your bag. Thank you very much.

- The Trust Game -

Before the main game, you will play a short game called the 'Trust Game'. In this game, you will be paired with another participant from this session, and you will face **two decision situations. You will only be paid for one of the two situations;** which one will be determined at random**.**

In the **first situation**, you are player 1. Both player 2 and you receive 10 points. **You**, as player 1, can choose to send none, some or all points to player 2. Once you have chosen the amount to send to player 2, this amount will be **tripled**: player 2 will receive three times the amount you sent. Player 2 will then decide how many of the points they received they will send **back to you**.

Example:

1. *Player 1 and player 2 both receive 10 points*
2. *Player 1 sends 5 points to player 2*
3. *Player 2 receives 5 x 3 = 15 points*
4. *Player 2 sends 7 points back to player 1*
5. *Player 2 has a total payoff of 10 + 15 - 7 = 18 points, and player 1 has a total payoff of 10 – 5 + 7 = 12 points.*

In this example, player 1 has made 7 – 5 = 2 points profit from sending points. However, you can also chose to send no points, and separately, player 2 can decide to send nothing back.

In the **second situation**, you are player 2 and another subject of this session is player 1. As player 2, you will start with 10 points. You will receive points from player 1 which will be tripled, and you decide if you want to send none, some or all of the points back to player 1.

The total amount of points for **one of these situations** will be multiplied by **30** and added to your final payoff at the end of the experiment.

- How it looks on your computer –

Below, you see the first screen where you play as player 1 and have to choose if and how many points you will send to player 2.

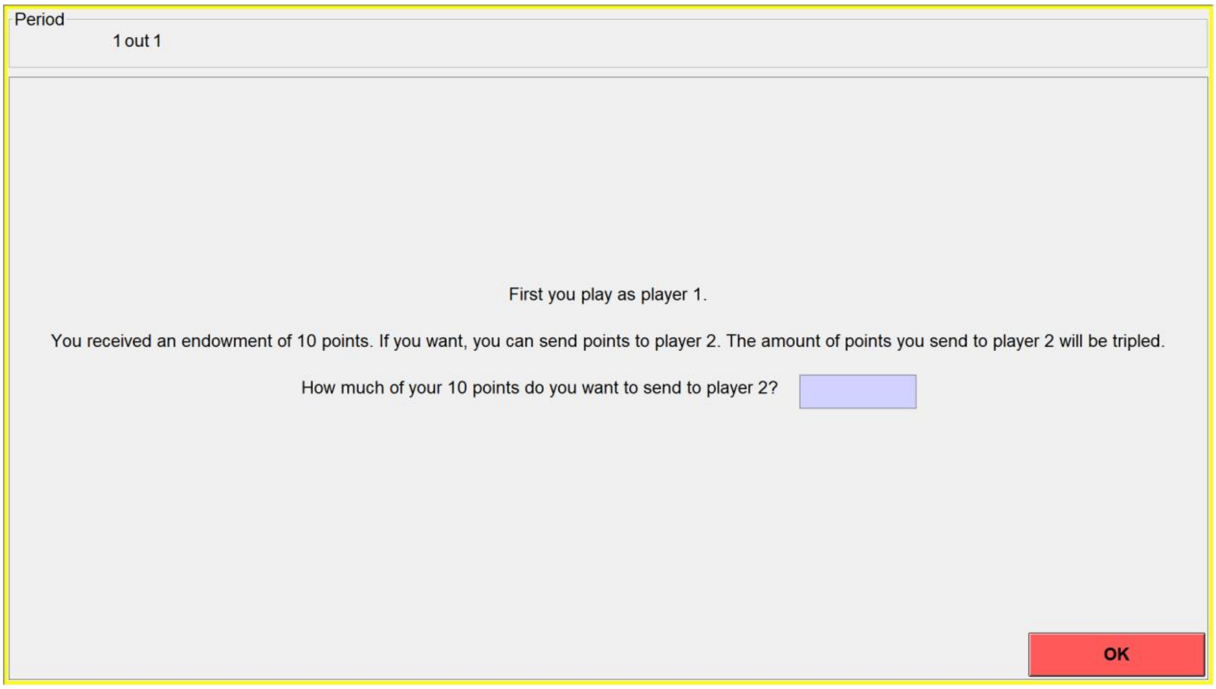

Below, you see the second screen, where you play as player 2. Here, you have to decide how many points you would send back if you received a certain amount of points from player 1:

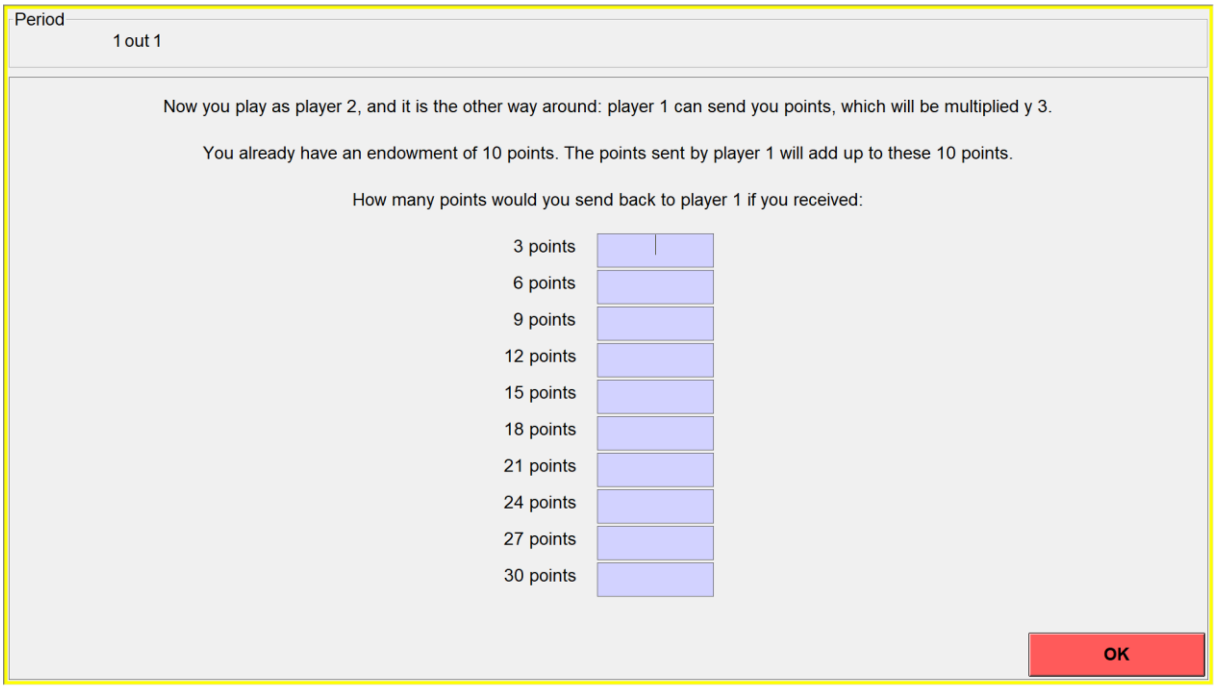

# - The fishing game -

We will now describe the decision situation in which you are placed.

You will play **three rounds** of this game to practise.

You are placed in a group consisting of you and **three other players** in this laboratory, with whom you will interact. Every player receives a budget of **50 points** in each period.

Imagine you and the others in your group are living in a village at a lake and for food you depend on the fish in this lake. Every period, you are asked how many of your budget points, between **0 and 50**, you want to invest in fishing from the lake.

For every point you invest, you catch fish. How many fish you catch depends on the number of fish in the lake.

Initially, the lake contains **600 fish**. With 600 fish in the lake, you catch one fish with a value of **4 points**, for every point you invest. You, thus, gain 3 points for every invested point (4 profit minus 1 invested point). In general, your earnings depend on the number of fish in the lake and are equal to $\frac{\text{the number of fish in the lake}}{150}$ per invested point, see the following table:

Fish in the lake	Earnings per point invested	Gains (earnings – investment) per point invested
600	4	3
450	3	2
300	2	1
150	1	0

Example: imagine there are 400 fish in the lake and you invest 15 points. Then you keep 35 points from your budget and you earn 15$\times \frac{400}{150}$ = 40 points (= 10 fish) from your investment. You end this period then with 35 + 40 = 75 points. To make things a little easier, you can always see on the screen how many points you will earn per invested point.

Fishing reduces the number of fish in the lake, but the lake will also recover to some extent every period. The number of fish taken out of the lake equals $\frac{fish\ in\ the\ lake \times total\ points\ invested\ in\ fishing}{600}$. So, if there are 600 fish in the lake, for every point invested, one fish is taken out; if there are, e.g., only 300 fish in the lake, for every point invested only half a fish is taken out of the lake. After each period, the fish population recovers again with an increase of 25%, but the lake can never contain more than 600 fish. See the following table.

Starting amount of fish	Amount invested per subject	Amount invested by all subjects together	Number of fish caught	Amount before recovery	Amount after recovery
600	*50*	*200*	*200*	*400*	*500*
600	40	160	160	440	550
**600**	**30**	**120**	**120**	**480**	**600**
600	20	80	80	520	600
300	50	200	100	200	250
300	40	160	80	220	275
300	30	120	60	240	300
300	20	80	40	260	325

For example (see the row in **bold**), if all four players in your group invest **30** in fishing, your group will invest 4 $\times$ 30 = 120 in total. This will decrease the number of fish in the lake to 600 – 120 = 480. Due

to increase of the fish in the lake, the number of fish in the lake will be 480 × 1.25 = 600 again in the next period. However, if all four players invest *50* (the row in *italics*), the number of fish in the lake will decrease with 4 × 50 = 200, which results in 600 – 200 = 400 fish. Due to the increase of the fish in the lake, the number of fish in the lake will be 400 × 1.25 = 500 in the next period. The number of fish in the lake, and with it the number of fish you can catch per invested point in fishing, will then decrease. To summarize, some important properties of fishing need to be considered:

- **The more fish in the lake, the more points you earn with the same investment of points**
- **If everyone invests 30 (or less) in fishing, the number of fish in the lake remains the same (or increases to a maximum of 600) over time**
- **If everyone invests more than 30 in fishing (or the total investment in fishing is more than 120), the number of fish in the lake decreases over time.**

- How it looks on your computer –

Below, you see the screen in which you make a decision of how many points (0 to 50) you want to invest in fishing. On the top of the screen, you see how many points you and your group as a whole invested in fishing in the previous periods.

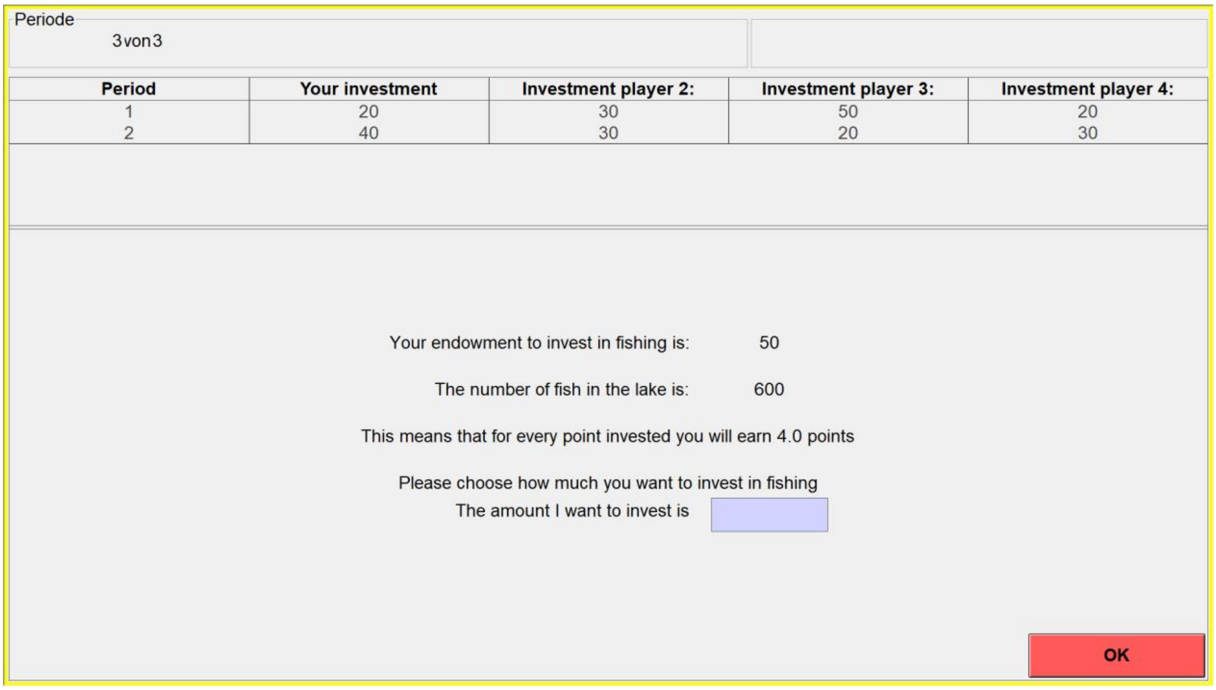

Below, you see the screen in which you can see your starting budget, invested points in fishing, earnings from that investment and your total earnings from this period.

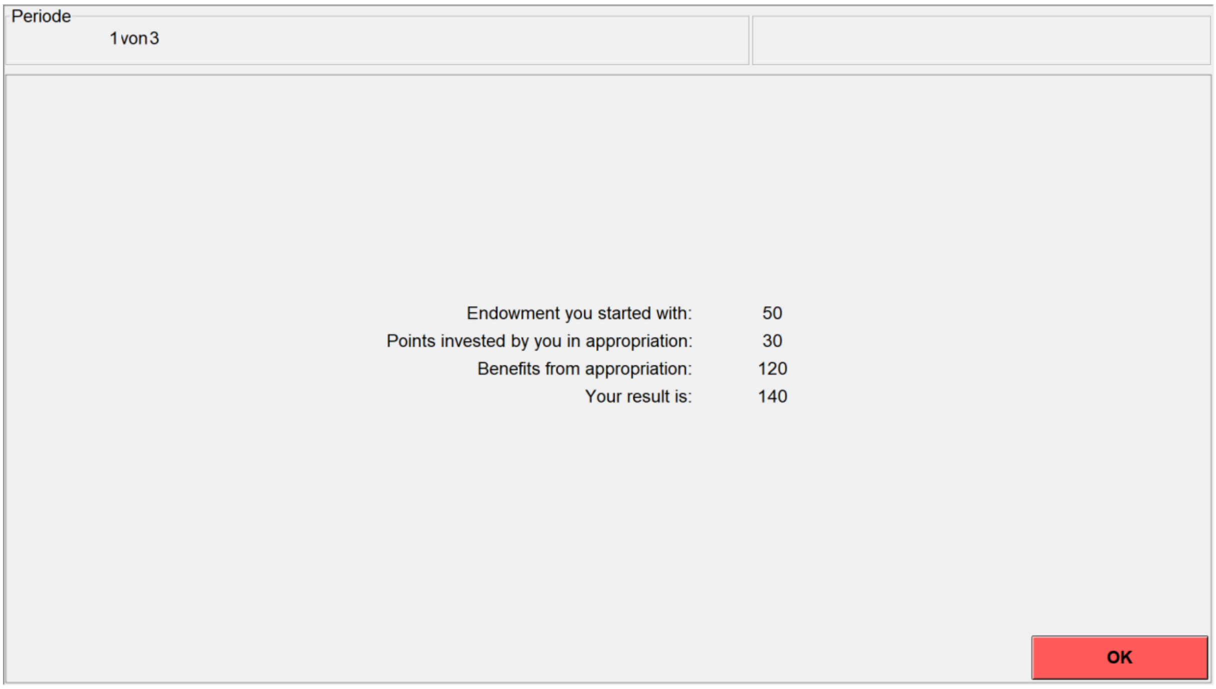

# - Identities -

In this part of the experiment, you will first be asked to express your preference for a few sets of paintings from the artists Paul Klee and Wassily Kandinsky. Based on your preferences, you will be labelled as part of the “Klees” or the “Kandinskys”.

Quiz

Together with your allocated group (Klees or Kandinskys), you will play a short quiz in which you have to guess who the painter is for several paintings that are shown.

If your group has **more right answers** than the other group, everyone in your group will earn 100 extra points.

If the **majority of the answers in your group** is right, everyone in your group will earn 100 extra points.

Division game

Before the fishing game continues, you play 3 short games in which you divide points between Klees and Kandinskys. One of these games will be randomly selected to be paid: the points will be added to a Kandinsky and a Klee.

Trust Game

After the quiz and the division game, you will play the trust game again: **once with a member of your group, and once with a member of the other group.**

<u>All the extra point</u>s from this part of the experiment will be added to your total payoff at the end of the experiment.

- How it looks on your screen -

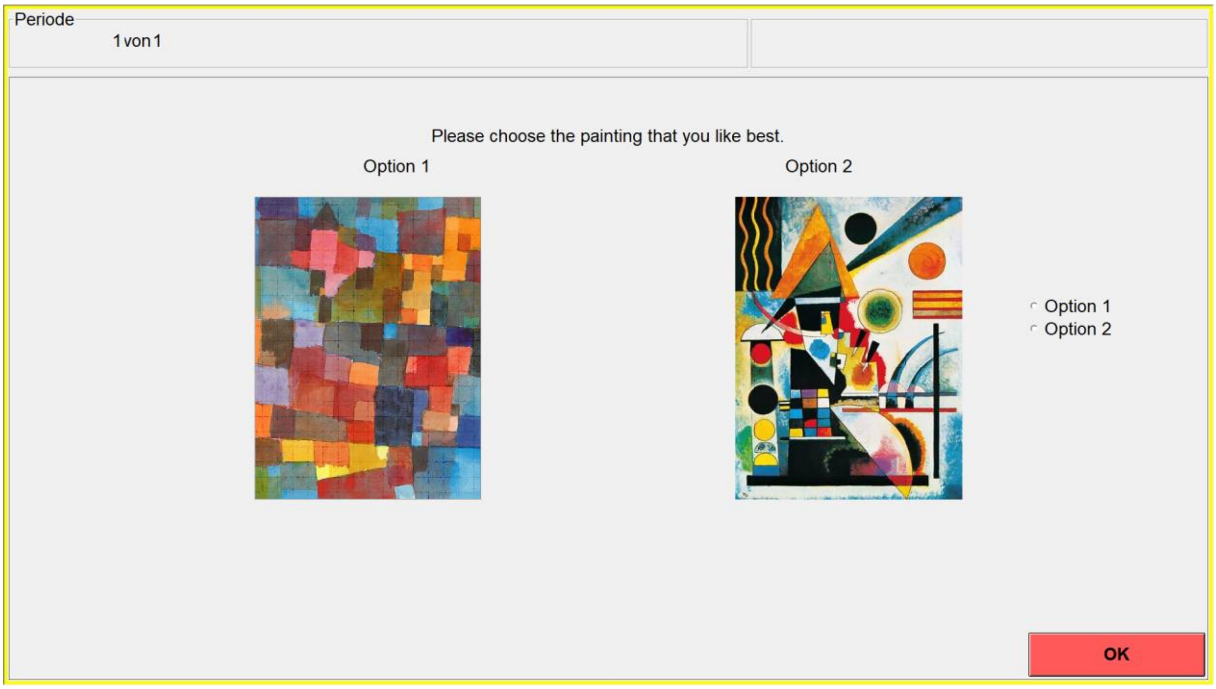

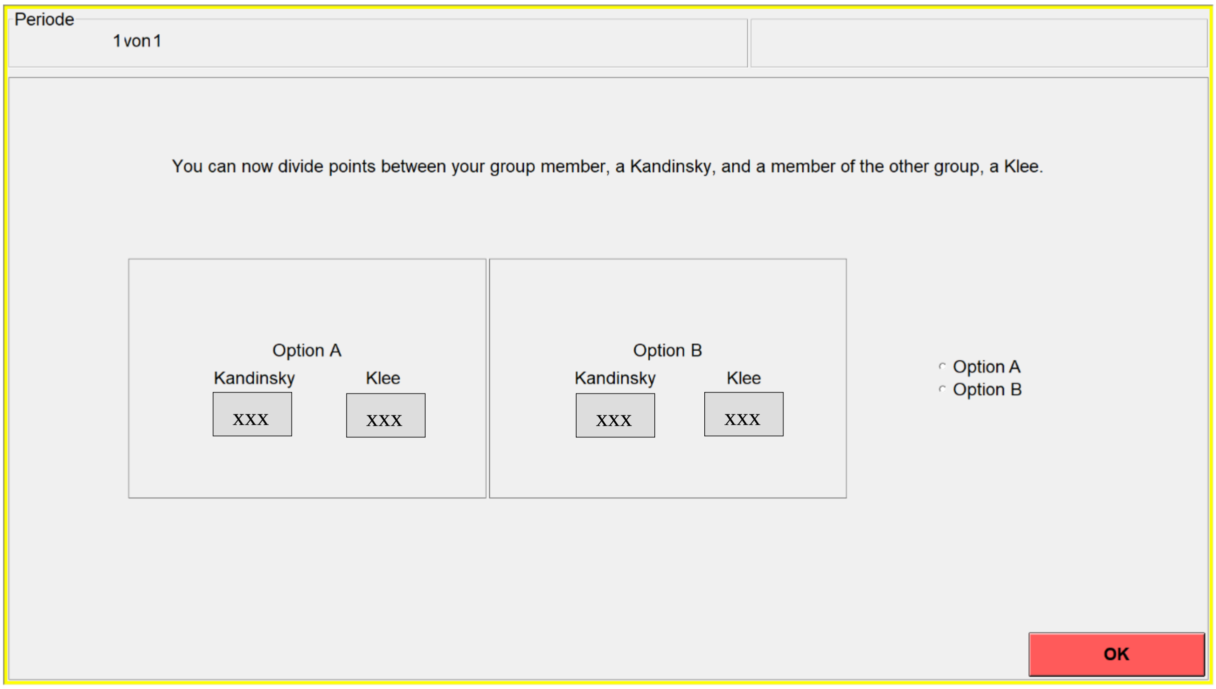

Supplement: S1 Text — The general experiment instructions that subjects receive upon entering the lab. (PDF) [file pone.0237870.s001.pdf]
