# Supplementary material for: Playing nice in the sandbox: On the role of heterogeneity, trust and cooperation in common-pool resources
Source: PLoS One. 2020 Aug 28;15(8):e0237870. doi: 10.1371/journal.pone.0237870 (PMC7454994; doi:10.1371/journal.pone.0237870)
Supplement: S2 Text — Treatment-specific instructions for subjects in the different treatments. Treatments are indicated on the top left of the page with an ‘E’ for economic heterogeneity, ‘S’ for sociocultural heterogeneity, ‘B’ for economic and sociocultural heterogeneity (‘Both’) and ‘N’ for no heterogeneity (the homogeneous control group). (PDF) [file pone.0237870.s002.pdf]

- Inequality –

When the Fishing Game starts, some of the players in your group have a **larger endowment** than others, which means they have the possibility to invest more points in fishing. You will be informed on the endowments of each player in your group. The situation will stay the same throughout **all periods** of the fishing game.

Note that the renewal rate of the fish in the lake **stays the same**. So even though some players have more points to invest in fishing, investing more than **120** points in total as a group will lead to a **decrease** in both the number of fish in the lake and the returns per invested point.

Lastly, a questionnaire will be started after which you will receive your payment.

S

- Different identities–

When the Fishing Game starts, you are placed in a group consisting of you and three other players in the laboratory, with whom you will interact. Two of the players will be Klees and two will be Kandinskys. You will know how much each Klee and each Kandinsky invests in fishing.

Lastly, a questionnaire will be started after which you will receive your payment.

- Different identities & Inequality –

When the Fishing Game starts, some of the players in your group have a **larger endowment** than others, which means they have the possibility to invest more points in fishing. You will be informed on the endowments of each player in your group. The situation will stay the same throughout **all periods** of the fishing game.

Two of the players will be Klees and two will be Kandinskys, and either the Klees or Kandinskys have a higher endowment than the other. You will know how much each Klee and each Kandinsky invests in fishing.

Note that the renewal rate of the fish in the lake **stays the same**. So even though some players have more points to invest in fishing, investing more than **120** points in total as a group will lead to a **decrease** in both the number of fish in the lake and the returns per invested point.

Lastly, a questionnaire will be started after which you will receive your payment.

-Same identities-

When the Fishing Game starts, you are placed in a group consisting of you and three other players in the laboratory, with whom you will interact. You will be placed in a group with 3 players of your own group (all Klees or all Kandinskys). You will know how much each person invests in fishing.

Lastly, a questionnaire will be started after which you will receive your payment.
