# Supplementary material for: Playing nice in the sandbox: On the role of heterogeneity, trust and cooperation in common-pool resources
Source: PLoS One. 2020 Aug 28;15(8):e0237870. doi: 10.1371/journal.pone.0237870 (PMC7454994; doi:10.1371/journal.pone.0237870)
Supplement: S3 Text — A description of all the characteristics of the Investment Game. (PDF) [file pone.0237870.s003.pdf]

## S3. Characteristics of the Investment Game

There are many variations on the Investment Game. In this version of the game, choices were made with regard to the following characteristics.

(1) Players will play the role of sender as well as the role of receiver once. Burks, Carpenter and Verhoogen [1] found that letting the players play both roles takes away a feeling of guilt that subjects in the sending role would otherwise experience towards the receivers, since the payoff would rely on only one interaction However, they also find that playing both roles reduces mutual trust and reciprocity. In this experiment, players play both roles but will be paid for only one. However, since subjects do not know for which interaction they will receive their payment, I do not expect the players' behaviour to be affected by feelings of guilt. An advantage of letting subjects play both roles is that more data on trusting and trustworthiness can be gathered, and different types of players - such as altruists, egoists and conditional co-operators - can be identified (see also [2]).

(2) Real players are used instead of computerised counterparts. If subjects suspect or know that their counterpart in an interaction is computerised, they will behave differently in the sense that they will send less money to the receiver [3, 4]. The meta-analysis of Johnson and Mislin [5], comprising 162 replications of the Investment Game, shows that playing against a real counterpart has a positive effect on trusting behaviour e.g. playing with a real person will yield higher amounts of points sent by the sender.

(3) A form of random payment is introduced, as stated earlier, in the sense that all subjects will be paid for one out of in total two interactions; they will be paid either for the sending or the receiving role. Random payment is in general suggested to yield more risk-averse behaviour from subjects, resulting in lower amount of points sent by the sender [6]. The meta-analysis of Johnson and Mislin [5] points out that there is indeed a negative effect of random payment on trust. However, this random payment is defined as only a subset of subjects receiving payment, while in the current experiment all subjects will be paid; the randomness lies in which role interaction will be paid. Based on the latter, subjects are not expected to be influenced by random payment.

(4) The strategy method is used, meaning that all subjects in the receiving role interaction have to indicate how much they would return to the sender for every possible amount of points received [7]. Some research suggests that providing this choice to subjects may alter their perception of the game [8, 9]. The research, on the other hand, suggests that the strategy method has no influence of subjects' behaviour [10]. The meta-data analysis of Johnson and Mislin [5] found no significant effects of this method on trust or trustworthiness.

(5) Subjects in the receiving roles will receive an endowment. This will cancel out the possible effect that inequity may have on subjects. If only the sender starts with an endowment, this may cause the sender to send money to the receiver out of a feeling of injustice or guilt instead of trust [11, 12]. If both players start with the same endowment, the act of sending money to the receiver can still increase the payoff for both players, and sending money while the other player has money already will be a more defined act of trust in the other player. The meta-analysis of Johnson  Mislin [5] shows no persistent negative effect of receiver endowment (only one out of three models on trust shows a significant negative effects of receiver endowment).

(6) We will use anonymity amongst subjects. Subjects will not see each other's decisions, not do they know with whom they are matched for the payoff interaction. This will prevent reputation (Kreps, 1990) and/or reciprocity of kind acts [13] from having an effect on trust and trustworthiness, enabling us to measure trust and trustworthiness without the shadow of the future nor from the past. The meta-analysis of Johnson and Mislin [5] shows weak support of the suggestion that anonymity has a

negative effect on trust.

## References


1. Burks S V, Carpenter J P, Verhoogen E. Playing Both Roles in the Trust Game. Journal of Economic Behavior & Organization. 2003;51(2):195–216.
2. Burks S, Carpenter J, Goette L. Performance Pay and Worker Cooperation: Evidence from an Artefactual Field Experiment. Journal of Economic Behavior & Organization. 2009;70(3):458–469.
3. Bottom WP, Holloway J, Miller GJ, Mislin A, Whitford A. Building a Pathway to Cooperation: Negotiation and Social Exchange between Principal and Agent. Administrative Science Quarterly. 2006;51(1):29–58.
4. Sanfey AG, Rilling JK, Aronson JA, Nystrom LE, Cohen JD. The Neural Basis of Economic Decision-Making in the Ultimatum Game. Science. 2003;300(5626):1755–1758.
5. Johnson ND, Mislin AA. Trust Games: A Meta-Analysis. Journal of Economic Psychology. 2011;32(5):865–889.
6. Bottom WP. Negotiator Risk: Sources of Uncertainty and the Impact of Reference Points on Negotiated Agreements. Organizational Behavior and Human Decision Processes. 1998;76(2):89–112.
7. Bahry D L, Wilson R K. Confusion or Fairness in the Field? Rejections in the Ultimatum Game under the Strategy Method. Journal of Economic Behavior & Organization. 2006;60(1):37–54.
8. Güth W, Huck S, Müller W. The Relevance of Equal Splits in Ultimatum Games. Games and Economic Behavior. 2001;37(1):161–169.
9. Roth AE. Bargaining Experiments. In: Kagel JH, Roth AE, editors. Handbook of Experimental Economics. Princeton, NJ: Princeton University Press; 1995. p. 253–348.
10. Brandts J, Charness G. Hot vs. Cold: Sequential Responses and Preference Stability in Experimental Games. Experimental Economics. 2000;2(3):227–238.
11. Adams JS. Inequity In Social Exchange. Advances in Experimental Social Psychology. 1965 Jan;2:267–299.
12. Adams JS, Freedman S. Equity Theory Revisited: Comments and Annotated Bibliography. Advances in Experimental Social Psychology. 1976 Jan;9:43–90.
13. Gouldner AW. The Norm of Reciprocity: A Preliminary Statement. American Sociological Review. 1960;25(2):161–178.
