# Supplementary material for: Playing nice in the sandbox: On the role of heterogeneity, trust and cooperation in common-pool resources
Source: PLoS One. 2020 Aug 28;15(8):e0237870. doi: 10.1371/journal.pone.0237870 (PMC7454994; doi:10.1371/journal.pone.0237870)
Supplement: S4 Text — A table that shows the two options that subjects faced in each of the three scenarios in the other-other DG. (PDF) [file pone.0237870.s004.pdf]

## S4 Details of Other-Other Dictator Game

**Table 1. Other-Other Dictator Game scenario 1 (A1, B1), 2 (A2, B2) and 3 (A3, B3)**

| Option given for: | | | | | |
|---|---|---|---|---|---|
| Klees & Kandinskys | | Klees | | Kandinskys | |
| A1 | | B1 | | B1 | |
| Klee | Kandinsky | Klee | Kandinsky | Klee | Kandinsky |
| 330 | 330 | 345 | 355 | 355 | 345 |
| A2 | | B2 | | B2 | |
| Klee | Kandinsky | Klee | Kandinsky | Klee | Kandinsky |
| 420 | 420 | 440 | 445 | 445 | 440 |
| A3 | | B3 | | B3 | |
| Klee | Kandinsky | Klee | Kandinsky | Klee | Kandinsky |
| 320 | 320 | 300 | 280 | 280 | 300 |
