# Supplementary material for: Playing nice in the sandbox: On the role of heterogeneity, trust and cooperation in common-pool resources
Source: PLoS One. 2020 Aug 28;15(8):e0237870. doi: 10.1371/journal.pone.0237870 (PMC7454994; doi:10.1371/journal.pone.0237870)
Supplement: S5 Text — Ordinal logistic regression on two survey questions on trust in and trustworthiness of other players. (PDF) [file pone.0237870.s005.pdf]

S5 Ordinal Logistic Regression on trust

Table 1. Ordinal Logistic Regression on post-experimental measures of trust (Odds Ratios)

	(1) Trust in other players	(2) Subjective trustworthiness of others
EH	1.667^{+}	1.211
	(0.462)	(0.333)
SH	1.604^{+}	1.131
	(0.447)	(0.320)
EHSH	1.672^{+}	1.058
	(0.463)	(0.299)
**Controls**		
Final Profit	1.190^{***}	1.392^{***}
	(0.055)	(0.068)
Age	1.042^{**}	1.018
	(0.013)	(0.013)
Friends	0.967	0.819^{+}
	(0.013)	(0.084)
Game Theory Experience	1.095	0.884
	(0.227)	(0.185)
Female	1.634^{*}	1.105
	(0.331)	(0.226)
Student	1.211	0.598
	(0.448)	(0.230)
Netherlands	1.115	1.260
	(0.249)	(0.285)
Observations	341	341

Note: Standard errors in parentheses.
$^{+}p<0.1$; $^{*}p<0.05$; $^{**}p<0.01$; $^{***}p<0.001$
N = 341 due to 3 missing responses on sex
Tables produced with *Stargazer* [1]

References

1. Hlavac M. Stargazer: Well-Formatted Regression and Summary Statistics Tables. R Package Version 5.2.2.; 2018.
