# Supplementary figures and images for: Playing nice in the sandbox: On the role of heterogeneity, trust and cooperation in common-pool resources
Source: PLoS One. 2020 Aug 28;15(8):e0237870. doi: 10.1371/journal.pone.0237870 (PMC7454994; doi:10.1371/journal.pone.0237870)

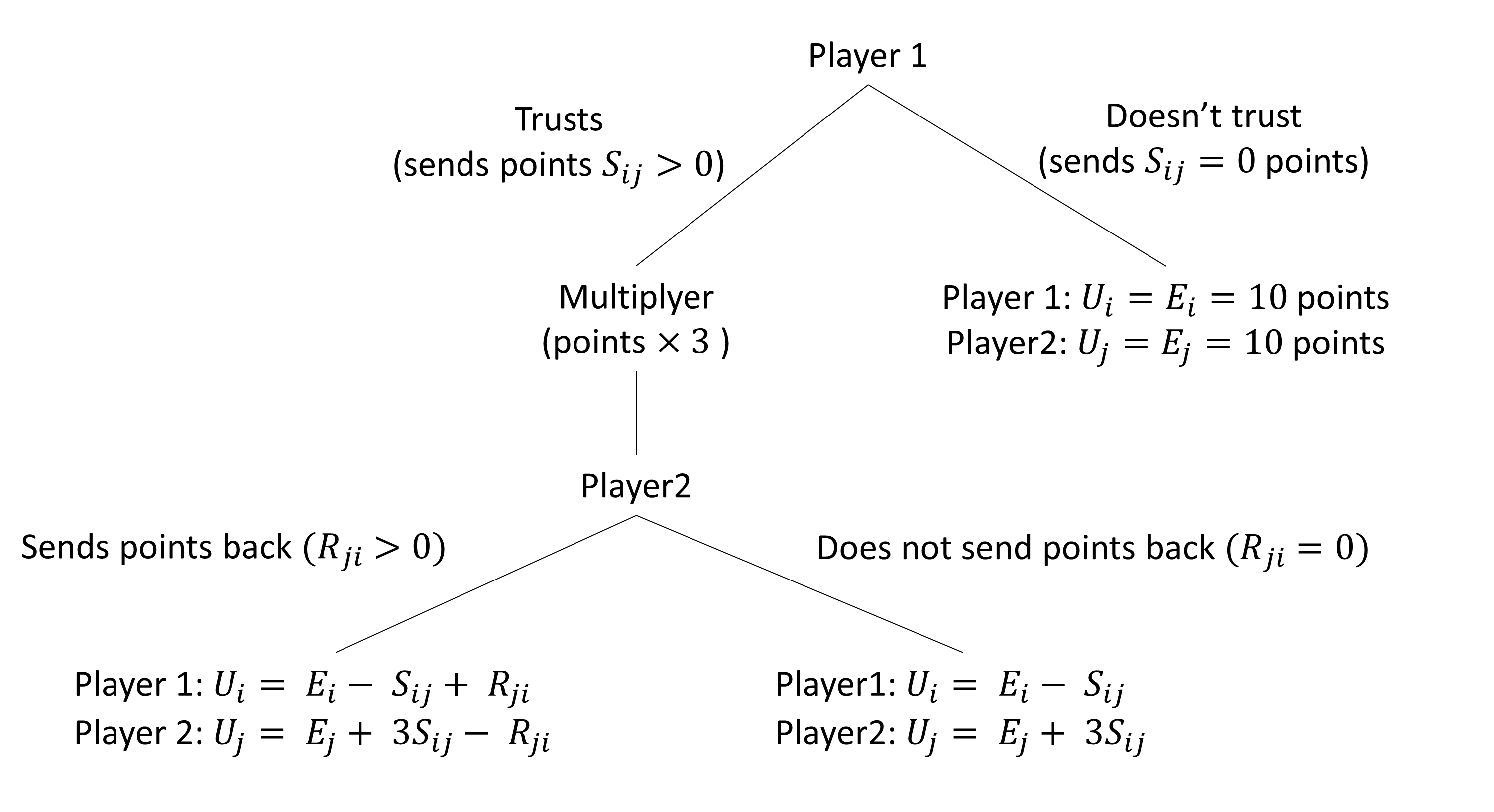

Supplement: S1 Fig — A graphic representation of the Investment Game as played in the current experiment. (TIF) [file pone.0237870.s006.tif]
